# Smoking cessation counseling in primary care settings: Cross-sectional views from patients and dentists as key stakeholders

**DOI:** 10.1371/journal.pone.0354366

**Published:** 2026-08-14

**Authors:** Shabnam Varmazyari, Pouya Bozorgi, Parvin Bastani, Mohammad Reza Khami

**Affiliations:** 1 Research Center for Caries Prevention, Dentistry Research Institute, Tehran University of Medical Sciences, Tehran, Iran; 2 School of Dentistry, Tehran University of Medical Sciences, Tehran, Iran; 3 Oral Health Department, Health Deputy, Shahid Beheshti University of Medical Sciences, Tehran, Iran; 4 Department of Community Oral Health, School of Dentistry, Tehran University of Medical Sciences, Tehran, Iran; Tarbiat Modares University Faculty of Medical Sciences, IRAN, ISLAMIC REPUBLIC OF

## Abstract

**Background:**

Despite attitudes’ central role in shaping health behaviors, little is known about the attitudes of dentists and patients, key stakeholders, toward smoking cessation counselling by dentists (SCCD), particularly within Iran’s primary care system. This study provides a side-by-side comparison of SCCD attitudes among these key stakeholder groups and examines their individual-level determinants.

**Materials and methods:**

This two-phase cross-sectional study, incorporating both descriptive and analytical elements, recruited dentists from all comprehensive healthcare centers (CHCs) in Tehran province via census (March–June 2019) and patients from three randomly selected CHCs in Tehran city (July–August 2020). Both groups completed questionnaires on SCCD attitudes and individual-level factors, with patient attitudes further analyzed across three dimensions: dentist competence, SCCD effectiveness, and oral symptoms as motivators. Statistical analyses included independent t-tests, multiple logistic regression, and simple and multiple linear regression.

**Results:**

Of 380 patients (68.9% response rate), 51.6% were female, with a mean age of 38.8 years (SD = 7.1). Among 180 dentists (93% response rate), 81.6% were female, with a mean age of 34 years (SD = 10.0). Among dentists, non-smokers (B = 5.99, p = 0.005) and those working in Tehran city (B = −3.24, p = 0.024) were more likely to show positive SCCD attitudes. Among patients, non-smokers (OR = 1.52, p = 0.049) and those with a smoking family member (OR = 0.61, p = 0.021) were more likely to deem SCCD effective. Employed patients saw oral symptoms as stronger quitting motivators (OR = 0.46, p = 0.001), and employed smoking patients were more willing to quit using SCCD (OR = 0.43, p = 0.010).

**Conclusions:**

Enhancing SCCD attitudes in Iranian primary care settings may be achieved through mentorship for smoking dentists, virtual or remote education for rural dentists, workplace-based SCCD for employed patients, and engagement in family and community advocacy for non-smoking patients.

## Introduction

Tobacco smoking exerts substantial harmful effects on the oral cavity. Among its most serious oral consequences is a substantially increased risk of oral squamous cell carcinoma in smokers compared to non-smokers [1]. Smoking is also a well-established contributor to periodontitis, poorer periodontal treatment outcomes, and subsequent tooth loss [2]. Beyond these conditions, it is associated with a range of adverse oral outcomes, including mucosal lesions, impaired wound healing, dental implant failure, and increased risk of dental caries [3–5]. The burden of tobacco use is disproportionately concentrated in low- and middle-income countries, where approximately 80% of the world’s tobacco users reside [6]. Iran is one such lower-middle-income country where oral disease burden is notably high among adults [7], an estimated 14% of adults regularly use tobacco [8], and tobacco smoking has been associated with higher rates of dental caries and oral mucosal lesions as well as impaired oral tissue repair [9–11].

These consequences underscore the need for effective, evidence-based tobacco smoking cessation interventions. The World Health Organization (WHO) recommends that healthcare providers routinely deliver brief smoking cessation advice to all tobacco users as part of standard care [12]. Oral health settings within primary care represent an important platform for tobacco cessation interventions, with the FDI World Dental Federation recommending that oral health practitioners integrate brief tobacco interventions into routine clinical and community practice [13,14]. Dentists are well positioned to contribute to cessation support because they can reach large numbers of tobacco users through routine dental visits, identify tobacco-related oral manifestations, use visible oral effects as motivation for quitting [3,14], with smoking cessation counseling provided by dentists (SCCD) shown to increase the likelihood of quitting compared to usual care [15]. In addition, primary care can be an effective platform for cessation support due to its accessibility, continuity of care, and sustained patient–provider relationships [16].

Despite this, SCCD implementation remains inconsistent in both dental and primary care settings [17,18]. This implementation gap is particularly relevant in the case of Iran, where scaling up brief cessation advice in primary care is a WHO-United Nations Development Program investment priority [16], and where an internationally recognized, well-established primary care system provides extensive urban and rural coverage [19]. Within this system, dentists contribute not only to clinical care but also to prevention, workforce supervision, surveillance, and community outreach [20]. Despite this structural capacity, the provision of SCCD within Iranian primary care settings remains suboptimal [21].

One possible explanation for this implementation gap is stakeholder attitudes. According to established behavioral frameworks, attitudes are key determinants of intention and subsequent behavior [22,23]. Thus, in the context of SCCD, attitudes toward the intervention may influence how counseling is offered, accepted, and perceived. Prior research has identified several attitudinal barriers to SCCD delivery among dentists, including concerns about negative patient reactions, low self-efficacy in delivering cessation support, and perceptions that smoking cessation falls outside their professional role [18,24–27]. From the patient perspective, reported attitudinal barriers include embarrassment, discomfort discussing smoking, fear of judgment, and skepticism regarding the effectiveness or relevance of dentist-delivered advice [18,24]. However, existing research has largely examined dentist and patient attitudes in isolation, limiting understanding of how they align or diverge. Moreover, few studies have explored multidimensional attitudinal constructs or examined how individual-level characteristics influence these attitudes. Research is particularly scarce on SCCD attitudinal barriers of providers and patients in Iranian primary care dental settings, with one existing study reporting a mix of knowledge, training, organizational, and structural barriers, but without a dedicated focus on attitudinal ones [21].

Therefore, the present study aimed to (1) compare attitudes toward SCCD among samples of primary care dentists and patients in Iran, while outlining key patient attitudinal domains, including perceptions of dentist competence, SCCD effectiveness, and oral symptoms as motivators for quitting, and (2) explore individual-level determinants of attitudes in both groups. By integrating dual-stakeholder perspectives within a primary care framework, this study seeks to generate insights that inform SCCD delivery in Iran and similar resource-constrained settings.

## Materials and methods

This manuscript was written in line with the STROBE checklist.

### Study design and ethics

This cross-sectional study incorporated both descriptive comparison of stakeholder attitudes and analytical modeling to examine associations between individual-level factors and SCCD attitudes. It was carried out in compliance with the Helsinki declaration and carried out in two phases with approval from the Tehran University of Medical Sciences Ethics Committee. In the first phase which took place between March 10th and June 30th 2019, dentists working in CHCs across Tehran Province were invited to participate (ethics code: IR.TUMS.DENTISTRY.REC.1397.174). Building on this, the second phase took place between July 1^st^ and August 30th 2020, when data were gathered from dental patients visiting CHCs in Tehran Province (ethics code: IR.TUMS.DENTISTRY.REC.1399.018). Written informed consent was obtained from both groups following the explanation of research objectives and putting emphasis on voluntary participation, participant anonymity, and data confidentiality. It was also pointed out that completing the questionnaire meant informed consent to participate. Consent for publication was also obtained.

### Setting

Iran’s primary healthcare system is organized through a tiered network in which village-based Health Houses provide first-level services, while rural and urban Comprehensive Health Centers (CHCs) serve as key primary care facilities at higher levels of care. CHCs have a central role in delivering preventive, educational, family health, vaccination, and environmental health services, and provide broad urban and rural population coverage [19,20].

For the dentist sample, all CHCs in Tehran province served as the study setting. Tehran province was selected because of logistical feasibility, high population density, and the inclusion of Tehran city, the capital of Iran.

For the patient sample, three CHCs in Tehran province were randomly selected using a random number generator. The selected CHCs were *Farmanfarmaian* on *Azarbayjan Street* and *Ayat* and *Imam Hassan Mojtaba* in the *Khaniabad* neighborhood.

### Sample

Eligible participants included dentists working in CHCs across Tehran Province during the study period, and dental patients aged 18 and over attending CHCs in Tehran City. Patients aged 18 years and older were included because 18 is the legal age of adulthood in Iran, permitting independent informed consent without parental or guardian involvement. In addition, inclusion of underage individuals would have introduced developmental and behavioral considerations specific to adolescent smoking and oral health that were beyond the scope of the present study. Therefore, restricting the sample to adults ensured ethical appropriateness and conceptual consistency.

For patients, the minimum required sample size was estimated to be 367 using the results of a pilot study (with a sample of 30) with a confidence interval of one proportion option in PASS 11 software, considering α = 0.05 (probability of type I error), p= 0.65 (estimated proportion of the desired trait), and d = 0.1 (acceptable error in estimating the desired ratio). Due to the selected centers’ comparable patient loads and patient demographic compositions, the research team decided that equal numbers of participants would be recruited from each center. To account for potential non-response and ensure adequate power, slightly more participants were recruited, resulting in 380 completed questionnaires.

For dentists, an estimate of the minimum required sample size for the multiple linear regression was conducted for 7 potential explanatory variables with a target power of 80%, a two-tailed alpha of 0.05, and the ability to detect a small-to-medium effect size (Cohen’s f^2^ = 0.075, corresponding to an R^2^ of 0.07), a minimum required sample size of 163 dentists was calculated to ensure adequate power. However, the total number of dentists working in CHCs in Tehran province at the time of the study was 188. Given this relatively small and finite target population, a census approach was adopted to enhance representativeness, minimize sampling bias, and maximize statistical power by inviting all eligible dentists to participate.

### Procedure

After coordination with the CHC managers, one of the investigators personally visited the designated centers. The investigator explained research objectives and, as outlined previously, obtained written informed consent from the participants. The investigator then distributed the questionnaires and was available to clarify any ambiguities, and also to collect the completed questionnaires. The procedure was identical for dentists and patients.

### Instrument and measures

The research instruments for dentists and patients were self-administered Persian questionnaires consisting of two sections addressing individual-level factors and attitudes toward SCCD (English translations are provided in Supplementary Files 1 and 2). The attitude items for both of these questionnaires were adapted from a previously validated questionnaire developed for primary care dental settings [21]. In the original study [21], the questionnaire underwent a validation process prior to its use. Face validity was examined by a group of 10 dentists together with an expert panel consisting of six community oral health specialists and one epidemiologist, who evaluated the clarity, wording, response formats, and overall readability of the items. Content validity was subsequently assessed by the same panel through ratings of each item’s relevance, simplicity, clarity, and necessity using a four-point scale, resulting in a scale content validity index of 0.96. Reliability was evaluated using a test–retest approach in which the questionnaire was administered twice to a subset of participants with a two-week interval between administrations. Item-level agreement coefficients ranged from 60% to 97.5%.

Because the attitude items were adapted from an instrument that had previously undergone formal validation, the present study did not repeat the full validation process. Instead, the adapted questionnaires were piloted among 15 dentists and 15 patients from the target population to confirm their suitability for the study context and to ensure that the items were clear, relevant, and easily understood. Internal consistency was then recalculated for the adapted instruments using Cronbach’s alpha. The dentist questionnaire demonstrated good internal consistency (α = 0.874). For the patient questionnaire, internal consistency was excellent for the “SCCD effectiveness” domain (α = 0.940) and acceptable for the “oral symptoms as motivators” domain (α = 0.729), whereas the “dentist SCCD competence” domain showed limited internal consistency (α = 0.231). Therefore, findings related to this latter domain were interpreted cautiously.

The individual-level characteristics recorded for patients and dentists were treated as explanatory variables in the present study. For patients, these variables included age, gender, education, occupation, cigarette, hookah, and pipe smoking status, having a smoking family member, and willingness to quit using smoking cessation counseling delivered by dentists (SCCD). For dentists, the variables included age, gender, years since graduation, practice location, employment status at the community health center (CHC), cigarette, hookah, and pipe smoking status, and having a smoking family member (yes/no).

Questions exploring patients’ and dentists’ attitudes were used to construct the study outcome variables as described below.

### Patient SCCD attitudes

This construct represented patients’ overall perceptions of receiving SCCD. It was measured using 12 items rated on a 5-point Likert scale ranging from “strongly disagree” (1) to “strongly agree” (5). By summing the responses, the total score ranged from 12 to 60, with higher scores indicating more positive attitudes toward SCCD. This variable is referred to as overall patient SCCD attitudes. The construct comprised three domains:

A Dentist SCCD competence: This domain assessed patients’ perceptions of their dentist’s competence in providing smoking cessation counseling. It was measured using 7 Likert-scale items (items 1–7 of the main questionnaire in Supplementary File 1), each scored from 1 to 5, yielding a total score range of 7–35.
B SCCD effectiveness: This domain evaluated patients’ beliefs regarding the effectiveness of SCCD in helping them quit smoking. It was measured using 3 items (items 8–10 of the main questionnaire in Supplementary File 1), each scored from 1 to 5, producing a total score range of 3–15.
C Oral symptoms as motivators: This domain assessed the extent to which patients perceived oral symptoms such as yellowed teeth and bad breath as motivations for quitting smoking and engaging in cessation counseling. It was measured using 2 items (items 11–12 of the main questionnaire in Supplementary File 1), each scored from 1 to 5, with total scores ranging from 2 to 10.

Dentist SCCD attitudes: This construct represented dentists’ perceptions regarding the provision of SCCD, including perceived professional responsibilities, their role as health role models, perceived barriers to providing SCCD, the perceived importance of smoking cessation counseling, the need for further training, and confidence in delivering SCCD. The construct was measured using 16 items rated on 5-point Likert scales ranging from “strongly disagree” (1) to “strongly agree” (5). The summed score ranged from 16 to 80, with higher scores indicating more positive attitudes toward SCCD provision.

### Statistical analyses

Data entry and analysis were performed using SPSS software (version 24 SPSS for Windows). Descriptive statistics were reported as means and standard deviations for continuous variables and frequencies and percentages for categorical variables.

For each study group, the individual-level factors were categorized for analysis based on their frequency distributions and prior relevant literature [21,28]. For patients, age was grouped as <39 and ≥39 years; occupation as employed (including governmental, non-governmental, and self-employed) and unemployed (including housekeepers, retirees, and those without employment); education as non-academic (high school diploma or lower) and academic (bachelor’s degree or higher); and smoking status as non-smoker (never or former smoker) and smoker (current smoker in any form). For dentists, age was categorized as <35 and ≥35 years; years since graduation as <5 and ≥5 years; employment status at CHCs as governmental/contractual versus official/other; and smoking status as non-smoker versus smoker using the same definitions.

For patients, independent samples T-tests were used to compare mean attitude scores across individual-level factors. Multiple logistic regression (Enter method) was then used to examine associations between categorized attitude domains and individual-level variables. Attitude scores were dichotomized at their mean values to distinguish less favorable versus more favorable attitudes (below and above 41.3 for overall SCCD attitudes, below and above 24.8 for attitudes toward dentist SCCD competence, below and above 9.6 for attitudes toward SCCD effectiveness, and below and above 6.8 for attitudes toward oral symptoms as motivators). Willingness to quit using SCCD was analyzed among smoking patients only.

For dentists, simple linear regression analyses were first performed to identify variables associated with SCCD attitude scores. Variables with p < 0.20 were entered into a multiple linear regression model using the Forward method. Statistical significance was set at p < 0.05 for all tests.

## Results

The response rate for patients was 68.9%, as 380 patients completed the questionnaire. This population had an average age of 38.8 years (SD = 7.10) and comprised of 51.6% females. More than half of this population had never smoked, while 45.8% had at least one smoker in their family. Nearly 60% had an academic education. Among the 186 current-smoking patients, 72 (38.7%) reported willingness to quit using SCCD.

Moreover, out of the 188 dentists targeted for participation, 180 returned completed questionnaires, resulting in a 95.7% response rate. Most of this population were female (81.6%) and had a mean age of 34 years (SD = 10.0). Just over two-thirds (69.6%) had graduated within the past five years, and 13.2% reported smoking cigarettes, hookah, or pipes. Moreover, close to half (45.6%) worked in the city of Tehran (Table 1).

**Table 1:** Individual characteristics of primary care patients (n = 380) and dentists (n = 180) in Tehran, Iran.

Number (%)	Dentist category	Number (%)	Patient category	Characteristic
32 (18.4%) 142 (81.6%)	Male Female	196 (51.6%) 184 (48.4%)	Male Female	Gender
122 (70.9%) 50 (29.1%)	< 35 ≥ 35	239 (62.9%) 141 (37.1%)	< 39 ≥ 39	Age
112 (69.6%) 49 (30.4%)	< 5 years since graduation ≥ 5 years since graduation	150 (40.1%) 224 (59.9%)	Non-academic Academic	Education
125 (71.8%) 49 (28.2%)	Governmental service and contractual Official and other	130 (34.2%) 250 (65.8%)	Unemployed Employed	Occupation
23 (13.2%) 151 (86.8%)	Smoker Non-smoker	186 (48.9%) 194 (51.1%)	Smoker Non-smoker	Smoking status
45 (26.2%) 127 (73.8%)	Yes No	174 (45.8%) 206 (54.2%)	Yes No	Smoking family member

### Patient and dentist attitudes toward SCCD

To address the first study objective, descriptive analyses were conducted to compare overall and item-level SCCD attitudes between primary care dentists and patients.

On average, dentists scored 70.2% of their maximum possible SCCD attitude score (mean = 56.2, SD = 9.3), whereas patients scored 44% of their maximum SCCD attitude score (mean = 26.4, SD = 5.9).

**Fig 1:**
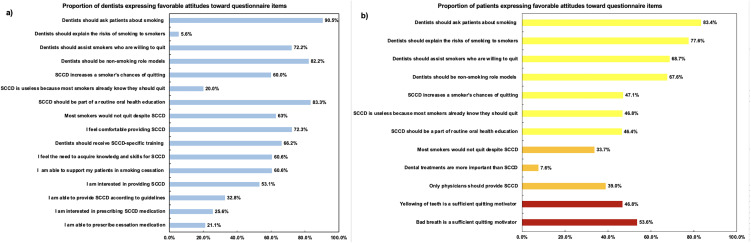
Proportions of primary care patients (n = 380) and dentists (n = 180) expressing favorable attitudes toward SCCD in Tehran, Iran. ^a^ Smoking cessation counseling by dentists. ^b^ In this figure, which illustrates item-level differences in attitudes between Tehran primary care a) dentists and b) patients, responses of ‘Agree’ and ‘Strongly agree’ to the attitude items were combined and classified as “Favorable”, while ‘Neutral,’ ‘Disagree,’ and ‘Strongly disagree’ were combined and classified as “Unfavorable”. Legend: a) Blue bars represent the percentage of dentists with favorable attitudes toward each item. B) Yellow, orange, and red bars depict patients’ favorable attitudes across different domains: yellow reflects perceptions of dentists’ competence in providing SCCD, orange reflects perceptions of SCCD effectiveness, and red reflects perceptions of oral symptoms as motivators for quitting.

Fig 1 illustrates both consensus and stark discrepancies in attitudes toward SCCD. Highest agreement was observed in the foundational step, with nearly all dentists (90.5%) and most patients (83.4%) expressing support for asking patients about smoking. However, the largest perceptual gap was exposed regarding the utility of providing information: only 5.6% of dentists agreed with explaining smoking risks, in sharp contrast to 77.6% of patients. This divide was further reflected in expectations, as two-thirds of dentists (63.0%) thought most smokers would not quit despite SCCD, compared with only one-third of patients (33.7%).

### Individual-level determinants of patients’ SCCD attitudes

To address the second study objective, regression analyses were conducted to identify individual-level determinants of SCCD attitudes among both stakeholder groups.

As demonstrated in Table 2, non-smoking patients were more likely to have positive attitudes toward SCCD effectiveness than smokers (OR = 1.52, p = 0.049) and patients without a smoking family member were more likely to have more negative views of SCCD effectiveness compared to those with a smoking family member (OR = 0.61, p = 0.021). Moreover, employed patients were more likely to consider symptoms as motivators for quitting (OR = 0.46, p = 0.001) than unemployed ones.

**Table 2:** Associations between SCCD^†^ attitudes and individual-level factors among primary care patients in Tehran, Iran (n = 380).

Characteristic	Characteristic category	Patient attitude domains	Odds Ratio (95% Confidence Interval)	P-value^*^
Gender	Female^¶^ Male	Overall patient SCCD attitudes	0.88 (0.58 to 1.33)	0.549
		Dentist SCCD competence	0.90 (0.59 to 1.36)	0.604
		SCCD Effectiveness	0.98 (0.64 to 1.48)	0.908
		Oral symptoms as motivators	0.78 (0.50 to 1.19)	0.249
Age	< 39^¶^ ≥ 39	Overall patient SCCD attitude	0.98 (0.64 to 1.50)	0.936
		Dentist SCCD competence	1.14 (0.74 to 1.75)	0.550
		SCCD Effectiveness	0.96 (0.63 to 1.48)	0.870
		Oral symptoms as motivators	0.88 (0.57 to 1.37)	0.573
Education	Non-academic^¶^ Academic	Overall patient SCCD attitudes	0.71 (0.47 to 1.08)	0.112
		Dentist SCCD competence	0.70 (0.46 to 1.07)	0.101
		SCCD Effectiveness	0.74 (0.49 to 1.13)	0.170
		Oral symptoms as motivators	0.93 (0.60 to 1.45)	0.763
Occupation	Employed^¶^ Unemployed	Overall patient SCCD attitudes	0.78 (0.50 to 1.21)	0.267
		Dentist SCCD competence	0.79 (0.51 to 1.23)	0.298
		SCCD Effectiveness	1.04 (0.67 to 1.62)	0.855
		Oral symptoms as motivators	0.46 (0.29 to 0.73)	0.001^*^
Smoking status	Smoker^¶^ Non-smoker	Overall patient SCCD attitudes	1.02 (0.67 to 1.54)	0.937
		Dentist SCCD competence	0.73 (0.48 to 1.20)	0.149
		SCCD Effectiveness	1.52 (1.00 to 2.31)	0.049^*^
		Oral symptoms as motivators	0.88 (0.57 to 1.36)	0.561
Smoking family member	Yes^¶^ No	Overall patient SCCD attitudes	0.73 (0.49 to 1.11)	0.140
		Dentist SCCD competence	0.87 (0.57 to 1.31)	0.499
		SCCD Effectiveness	0.61 (0.41 to 0.93)	0.021^*^
		Oral symptoms as motivators	0.66 (0.43 to 1.02)	0.061

^†^Smoking cessation counseling by dentists

^‡^Calculated using logistic regression

^¶^Reference category

* Significant findings with p-values of less than 0.05.

As shown in Table 3, among smoking patients, employed patients demonstrated more willingness to quit using SCCD (OR = 0.43, p = 0.010) compared to unemployed ones.

**Table 3:** Willingness to quit using SCCD† by individual-level factors among smoking primary care patients in Tehran, Iran (n=186).

Characteristic	Characteristic category	Odds Ratio (95% C.I)	P-value^*^
Gender	Female^¶^ Male	1.05 (0.56 to 1.97)	0.871
Age	< 39^¶^ ≥ 39	1.16 (0.61 to 2.18)	0.652
Education	Non-academic^¶^ Academic	0.85 (0.45 to 1.59)	0.607
Occupation	Employed^¶^ Unemployed	0.43 (0.22 to 0.82)	0.010*
Smoking family member	Yes^¶^ No	0.90 (0.49 to 1.66)	0.732

^†^Smoking cessation counseling by dentists

^‡^Calculated using multiple logistic regression

^¶^Reference category

*Significant findings with p-values of less than 0.05.

### Individual-level determinants of dentists’ SCCD attitudes

Bivariate associations between individual-level factors and dentists’ SCCD attitude scores are presented in the S1 Table. According to this, dentists who had graduated more than five years prior (MD = −3.0, p = 0.049), those working in Tehran city (MD = 3.6, p = 0.013), and non-smoking dentists (MD = −6.2, p = 0.003) demonstrated significantly more positive attitudes toward SCCD.

Next, multiple linear regression tests were conducted to continue addressing the second study objective. These tests demonstrated that non-smoking dentists were more likely to demonstrate positive SCCD attitudes than smoking dentists (B = 5.99, 95% CI: 1.84 to 10.13, p = 0.005) and dentists practicing in bordering towns were more likely to demonstrate negative SCCD attitudes than dentists in Tehran city (B = −3.24, 95% CI: −6.04 to −0.43, p = 0.024).

## Discussion

This study offers one of the first side-by-side examinations of primary care dentists’ and patients’ attitudes toward SCCD and their individual-level determinants within Iran’s LMIC context. Although dentists demonstrated strong conceptual endorsement of SCCD, their limited confidence and perceived implementation barriers contrasted with patients’ more moderate and cautious receptivity, pointing to an attitude–implementation gap among dentists and a perceptual misalignment between dentists and patients. Across both stakeholder groups, non-smoking status emerged as a consistent individual-level determinant of more favorable SCCD attitudes. Beyond this, patient SCCD attitudes were associated with employment and family smoking status, with employed patients showing greater responsiveness to oral symptoms as quitting motivators and those with smoking family members more likely to perceive SCCD as effective, while dentists’ attitudes were related to practice environment, with more favorable views observed in better-resourced urban settings.

Dentists in this study demonstrated strong overall endorsement of SCCD but reported limited confidence and perceived training deficits. Similar patterns have been observed in other settings, where dentists’ positive SCCD attitudes did not necessarily translate into implementation readiness [29]. This suggests that barriers are less attitudinal and more structural or capability-related, including insufficient training, limited organizational support, or competing clinical demands. In the Iranian primary care context, where dentists assume multiple roles within a resource-constrained system [30], these factors may be particularly relevant. In contrast, patients were generally open to discussing smoking but expressed skepticism regarding the effectiveness of SCCD and the dentist’s role in delivering it. This partially contrasts with more recent evidence from other dental-care settings, where patients generally expressed receptivity toward dentist-led cessation support, including willingness to be asked about smoking, receive cessation advice, and discuss cessation aids within dental care [24,26,31]. A plausible explanation could be the limited routine provision of SCCD within Iran’s oral healthcare system, which may have reduced patients’ exposure to the intervention and thus, decreased their familiarity with and confidence in such interventions [21].

Non-smoking status emerged as a consistent determinant of more favorable SCCD attitudes across both stakeholder groups. Among patients, non-smokers were more likely to perceive SCCD as effective, broadly aligning with dental-patient studies showing more favorable awareness or attitudes toward dentists’ smoking-cessation role among non-smokers than smokers [32,33], and potentially reflecting lower defensiveness and greater receptivity to anti-smoking messaging among non-smoking individuals. Similarly, non-smoking dentists demonstrated more positive SCCD attitudes, consistent with findings from Yemen and Saudi Arabia [28,34], and possibly reflecting reduced cognitive dissonance and stronger alignment between personal behavior and professional responsibilities. However, this pattern is not entirely consistent, as a more recent US study of dental and allied-dental faculty found no significant association between smoking status and 5 A’s confidence; instead, greater confidence was associated with lower perceived barriers and higher perceived effectiveness of cessation interventions [35]. This is relevant to the present findings because the dentist SCCD attitude score was not limited to general approval of counseling, but also incorporated closely related dimensions such as perceived professional responsibility, role-model perceptions, perceived barriers, perceived importance of SCCD, training needs, and confidence in delivering SCCD. Therefore, the observed association between non-smoking status and more favorable dentist SCCD attitudes may reflect not only smoking status itself, but also variation in these underlying attitudinal and capability-related components. An Australian study further complicates the pattern by showing that former-smoking oral health professionals reported more favorable attitudes toward cessation involvement than never-smokers [36]. Together, these findings suggest that provider smoking status may not operate uniformly across settings and that SCCD attitudes may also depend on more proximal factors, including perceived barriers, perceived effectiveness, confidence, professional-role perceptions, and whether former smokers are analyzed separately or grouped with never-smokers. Further research is needed to clarify how current, former, and never-smoking status relate to the different components of SCCD attitudes across settings.

Employment status emerged as a key determinant of patient SCCD attitudes. Employed smoking patients were more likely to express willingness to quit using SCCD, and employed patients were more likely to view oral symptoms as motivators for quitting. The greater willingness to quit among employed smokers may reflect increased salience of the functional and social consequences of oral health in workplace contexts, where appearance and interpersonal interactions are more prominent. Only indirect evidence supports the role of workplace context in shaping cessation behavior. For example, a Turkish workplace-based cessation study reported high quit success following structured occupational interventions [37]. However, contradictory evidence from a UK study suggests that workplace may also hinder cessation efforts by embedding smoking within social routines and stress-coping practices [15]. Given the lack of directly comparable evidence in SCCD or primary care settings, further research is needed to clarify how employment status shapes SCCD-related attitudes.

Employed patients being more likely to perceive oral symptoms, specifically the yellowing of teeth and bad breath, as quitting motivators, likely reflects the importance of esthetic and socially noticeable oral changes at the workplace. Evidence directly examining this relationship among employed individuals is limited. However, indirect evidence among dental patient populations exists that both supports and challenge it. The previously noted qualitative UK study among smokers with periodontitis supports this finding, showing that visible and personally experienced impacts, such as staining, tooth loss, and tooth mobility, can act as prompts for cessation [15]. A Swedish study found that oral symptoms like tooth discoloration and bad breath are common and seen as reasons to quit, but they were not linked to higher motivation to quit, while affection by was [38]. This suggests that perceptible oral symptoms may not equally impact patients’ reasoning around quitting in different contexts. However, the present and Swedish studies differed in their assessment of oral symptoms. The former focused on two specific perceptible symptoms as motivators, whereas the latter examined broader complaints and found only periodontal conditions significantly associated with cessation motivation, which may explain the discrepancy. Given the limited evidence available, the impact of oral symptoms on employed individuals’ quitting motivation warrants further investigation.

One relatively underexplored contextual finding was that patients with a smoking family member were more likely to perceive SCCD as effective. Although direct evidence is limited, this finding can be interpreted through broader literature on the impact of close social relationships on smoking. For example, the previously noted qualitative UK study reported that supportive family members facilitated quit attempts while smoking peers hindered them [15]. Similarly, a study of Turkish dental patients found smoker friends a primary barrier to quitting [33]. Drawing from these findings, exposure to family smoking in the present study may have increased awareness of the challenges of quitting and the limitations of unaided attempts, thereby making structured support such as SCCD appear more effective. However, as this interpretation is based on indirect evidence, further research is needed to clarify the relationship and its underlying mechanisms.

Finally, dentists practicing in Tehran demonstrated more favorable SCCD attitudes than those in rural or bordering areas, representing another context-specific and understudied finding. This aligns with broader evidence of stronger cessation engagement in urban settings. For example, an Indian study reported more positive attitudes and greater training among urban dentists compared to rural auxiliaries, alongside weaker organizational support in rural settings [39], while a U.S. study found lower receipt of cessation treatment in rural populations due to service delivery barriers [40]. Together, these findings suggest that the more favorable attitudes observed among Tehran-based dentists may reflect better access to training and organizational support, although this urban–rural divide warrants further investigation.

### Strengths and limitations

The present study has several limitations that should be considered when interpreting the findings. Its cross-sectional design precludes causal inference, and self-reported measures may introduce social desirability bias. Dentists were recruited by census and were predominantly female recent graduates, reflecting Iran’s Ministry of Health workforce allocation to urban primary care centers; as this group may be more health-conscious, their overrepresentation may limit generalizability and inflate positive SCCD attitudes. A temporal gap between dentist (2019) and patient (2020) data collection, due to COVID-19-related constraints and reduced dental attendance, may have influenced patients’ SCCD attitudes. Additionally, the dentist SCCD competence domain showed low internal consistency in the present sample, suggesting that the items grouped under this domain may not have captured a single homogeneous construct, possibly reflecting different aspects of perceived dentist role and competence. Considering the conceptual breadth of the domain and given the theoretical relevance of its aspects to SCCD counseling, we ultimately retained its items to preserve content validity. Nonetheless, findings related to this specific domain should be interpreted cautiously. Despite these limitations, the study makes a meaningful contribution by addressing an evidence gap in an understudied LMIC primary care context, parallel assessment of dentists and patients’ attitudes, and the inclusion of patient attitude domains.

Building on the present study’s findings, targeted strategies can be developed for improving SCCD attitudes in Iranian primary care settings and, with caution, comparable LMIC contexts. Among dentists, given that positive attitudes did not translate into implementation readiness, smokers may benefit from reflective, peer-based attitude-building approaches, while those in rural settings may require remote continuing education, virtual mentorship, and stronger organizational support to enhance confidence and delivery capacity. Among patients, the generally cautious and sometimes skeptical receptivity toward SCCD underscores the need to strengthen its perceived effectiveness and relevance; in this regard, the greater receptivity observed among employed individuals supports integrating workplace-based cessation initiatives that emphasize visible oral health consequences. Additionally, engaging non-smoking patients and family members as SCCD advocates may help reinforce more favorable attitudes toward SCCD among smokers and increase confidence in its effectiveness.

Future studies should examine how workplace contexts influence patient engagement or the viewing of oral symptoms as quitting motivators, how family smoking exposure shapes perceived SCCD effectiveness, and the mechanisms driving rural–urban differences in dentists’ attitudes. Future studies should further evaluate and refine the dentist competence subscale of the patient questionnaire before using it as a standalone measure. Finally, this research should be extended to other regions and populations to improve generalizability.

## Conclusions

This study examined attitudes toward SCCD among primary care patients and dentists in Tehran and identified their individual-level determinants. Overall, both groups recognized the role of dentists in SCCD; however, a clear gap emerged between dentists’ conceptual support and their perceived implementation readiness, alongside patients’ cautious views regarding the intervention’s effectiveness. Key individual-level determinants of SCCD attitudes included non-smoking status across both groups, employment status and family smoking among patients, and practice setting among dentists. Building on these findings, improving SCCD attitudes in primary care requires targeted approaches that address gaps in confidence and implementation capacity, particularly among smoking dentists and those practicing in rural or underserved settings, while also strengthening the perceived effectiveness and relevance of SCCD among patients, particularly employed individuals and those embedded in smoking-affected family environments. Future research should explore the mechanisms through which workplace, family, and practice environments shape these stakeholders’ attitudes toward SCCD.

## Supporting information

S1 File English translation of the questionnaire for patients. (DOCX)

S2 File English translation of the questionnaire for dentists. (DOCX)

S3 File Anonymized dataset of patients. (XLSX)

S4 File Anonymized dataset of dentists. (XLSX)

S1 Table The table presenting bivariate associations between individual-level factors and dentists’ SCCD attitude scores. (DOCX)
