# Supplementary material for: Smoking cessation counseling in primary care settings: Cross-sectional views from patients and dentists as key stakeholders
Source: PLoS One. 2026 Aug 14;21(8):e0354366. doi: 10.1371/journal.pone.0354366 (PMC13475981; doi:10.1371/journal.pone.0354366)
Supplement: Supplementary file 1 [file pone.0354366.s007.pdf]

Dear Dr. Zarei and Reviewers,

We sincerely thank you for your careful evaluation of our manuscript.

We appreciate the constructive feedback provided by the Academic Editor and both reviewers. We have carefully addressed each comment below. All changes have been incorporated into the revised manuscript and highlighted in the tracked version.

ACADEMIC EDITOR COMMENTS

Comment 1 (Academic Editor):

The introduction must be updated with recent references to reflect the modern epidemiological landscape (not only from the last 4 years but broadly recent literature).

Response:

We thank the Academic Editor for this important recommendation. The Introduction has been comprehensively revised to incorporate more recent epidemiological and contextual evidence. We prioritized the inclusion of up-to-date global and national data, particularly from recent WHO-aligned reports, national surveys, and contemporary peer-reviewed studies. Additionally, we strengthened the representation of Iran-specific evidence to better reflect the current epidemiological and health system context. We have also replaced several older foundational citations and supplementing retained ones with contemporary sources.

Change Made:

- Replaced earlier global burden framing with updated distribution statement:
- Added recent Iran-specific evidence:
    - STEPS 2021 prevalence data
    - Recent Iranian studies on oral mucosal lesions and caries among smokers (2024–2025).
- Updated oral health evidence base:
    - Added a 2020 systematic review on smoking and periapical disease.
- Shifted away from reliance on older WHO 2005-style epidemiology toward mixed recent sources.

Comment 2 (Academic Editor):

Clarify the study design (e.g., descriptive vs analytical).

Response: We thank the Academic Editor for this important clarification. This comment overlaps with Reviewer comments regarding study design specification. In response, we have explicitly clarified throughout the manuscript that the study adopts a cross-sectional design incorporating both descriptive and analytical components. Especially, we have clarified the distinction between descriptive findings and analytical interpretation by beginning with the framing of desrcritpve findings around and then also restructuring the Discussion around shared analytical determinant domains across stakeholders. In the previous version, findings were often followed by descriptive summaries of literature. In the revised version, each paragraph begins with a specific finding and proceeds to analytical interpretation within a determinant-based framework, supported by selectively integrated literature .

Change Made:

- Revised the Abstract to explicitly state: *"This two-phase cross-sectional study, incorporating both descriptive and analytical elements…"*
- Revised the Study design and ethics subsection to clarify: *"This cross-sectional study incorporated both descriptive comparison of stakeholder attitudes and analytical modeling…"*
- Refined the Introduction (final paragraph) to clearly distinguish between:
    - the comparative (descriptive) objective, and
    - the determinant-focused (analytical) objective.
- Added transition language in the Results section linking descriptive analyses to the first objective and regression analyses to the second.

Across the Discussion:

- Restructured to begin with descriptive study findings and gaps followed by analytical findings (the indibidual-level determinants)
- Shift implemented from stakeholder-based narration to determinant-based analytical synthesis
- Added interpretive synthesis statements across paragraphs

Comment 3 (Academic Editor):

Provide clearer details regarding the validity, reliability, and psychometric properties of the research instruments.

Response:

We appreciate this comment. This issue was also raised by Reviewer #1 (Comments 16–19) and Reviewer #2 (Comment 32). In response, we have expanded the description of the research instrument to provide a clear and transparent summary of its development, validation, and reliability assessment.

Change Made:

- Expanded the "Instrument and measures" section to include:

    - Face validity: assessed by 10 dentists and an expert panel (community oral health specialists and an epidemiologist), including evaluation of clarity and readability.
    - Content validity: assessed via structured ratings (relevance, simplicity, clarity, necessity), resulting in a content validity index of 0.96.
    - Reliability: described explicitly as a test–retest procedure with a two-week interval, with item-level agreement coefficients ranging from 60% to 97.5%.
- Added clarification that the instrument was piloted in the present study (n=15 dentists, n=15 patients) to ensure contextual appropriateness and clarity.
- Consolidated instrument description into a single, structured subsection to improve readability and avoid redundancy.

Comment 4 (Academic Editor):

Refine statistical reporting.

Response:

We thank the Academic Editor for this suggestion. This comment overlaps with Reviewer #1 Comment 22 and Reviewer #2 Comment 33. In response, the Statistical Analyses section has been revised to improve clarity, reduce redundancy, and align reporting with standard conventions.

Change Made (specific):

- Streamlined the Statistical analyses section by:
    - Removing repeated descriptive explanations already presented in Table 1.
    - Reorganizing variable categorization into a more concise and structured format.
- Clarified analytical procedures by explicitly distinguishing:
    - Descriptive analyses (means, SDs, frequencies)
    - Comparative analyses (t-tests)
    - Regression analyses (logistic and linear models)
- Improved clarity of regression reporting by:
    - Clearly defining outcome categorization thresholds
    - Separating patient and dentist analytical strategies
- Standardized terminology and improved readability by reducing overly detailed procedural language.

Comment 5 (Academic Editor):

Streamline the discussion to provide a more cohesive and rigorous contribution.

We thank the Academic Editor for this important comment. The Discussion has been fundamentally restructured to improve both cohesion and analytical rigor. In the previous version, the Discussion was organized in a largely segmented manner, with separate and partially

repetitive descriptions of dentist and patient perspectives, resulting in fragmentation and duplication .

In the revised version, we reorganized the Discussion around shared determinant domains of SCCD attitudes across both stakeholder groups**,** including smoking status, employment, and contextual factors (practice setting for dentists and family smoking for patients). This restructuring enabled a transition from parallel descriptive narration to an integrated framework in which findings are interpreted mechanistically across stakeholders. Redundant contrasts between dentists and patients were removed, and each paragraph now addresses a single determinant within a unified explanatory narrative.

Changes made:

- Global restructuring of Discussion:
  - From:
    - separate dentist and patient sections
  - To:
    - determinant-centered structure shared across stakeholders
- Opening paragraph rewritten:
  - Also an analytical framework (attitude–implementation gap + cross-stakeholder determinants)
- Determinant-based paragraphs created:
  - Non-smoking status
  - Employment
  - Context (practice setting + family smoking environment)
- Redundant dentist–patient contrast removed from mid-Discussion

Comment 6 (Journal Requirement):

Ensure manuscript meets PLOS ONE style requirements.

Response:
Thank you for this reminder. We have carefully reviewed the revised manuscript to ensure full compliance with PLOS ONE formatting and style guidelines. This included standardizing section structure, reference formatting, in-text citation style, figure and table presentation, and terminology in accordance with journal requirements. We also performed a thorough editorial review of the manuscript to ensure consistency, clarity, and adherence to PLOS ONE submission standards.

Comment 7 and 8 (Journal Requirement):

Funding information mismatch between "Funding Information" and "Financial Disclosure." Clarify role of funders in study design, data collection, analysis, decision to publish, or manuscript preparation.

Response:
Thank you for this query. As noted above, this study was supported through institutional funding from Tehran University of Medical Sciences (TUMS), for which no specific grant or award number is assigned. In this context, project identification and verification are linked to the study's ethics approval.

To ensure transparency, we have already provided the relevant ethics approval codes within the manuscript. In addition, the official ethics approval document—previously submitted as part of the supporting materials—clearly states the approval at the beginning of the document, followed by the corresponding ethics approval ID and detailed study specifications. These specifications (including study population characteristics) are consistent with those reported in the manuscript.

Change Made:

- Updated the **Funding statement in the cover letter** that read s:

"This study was supported in part by Tehran University of Medical Sciences (TUMS). The funder had no role in study design, data collection and analysis, decision to publish, or preparation of the manuscript."

Comment 9 (Journal Requirement):

Remove or anonymize identifying data from Supporting Information files.

Response:
Thank you for this important reminder. We have carefully reviewed all Supporting Information files and removed any potentially identifying information. All direct and indirect identifiers have been eliminated from both datasets. The revised Supporting Information files now include only variables that are directly relevant to the analyses and reported results presented in the manuscript.

Change Made:
All Supporting Information datasets (S3 and S4 Files) have been revised to remove any potential identifiers. Only de-identified variables corresponding to the study analyses have been retained.

REVIEWER #1 COMMENTS

Comment 10 (– Abstract):

Specify whether the cross-sectional study is descriptive or analytical.

Response:
Thank you for highlighting this point. The present study is a cross-sectional design incorporating both descriptive and analytical components. The descriptive component provides a structured comparison of SCCD attitudes between dentists and patients, while the analytical component examines associations between individual-level factors and attitude domains using regression modeling. To ensure clarity, we have revised the Abstract, Introduction, Methods, Results, and Discussion sections to explicitly specify both components of the study design and objectives.

Change Made:

- The study design statement in the Abstract and Methods sections now clearly indicates that the study combines descriptive comparison with analytical modeling.

- The Introduction and Discussion opening paragraphs have been refined to differentiate the comparative descriptive component from the analytical examination of determinants.

Comment 11 (– Abstract):

Keywords should be revised to match the title and abstract.

Response:
Thank you for this helpful suggestion. The keywords have been revised to better align with the terminology and core constructs emphasized in the title and abstract. Specifically, we ensured that the selected terms reflect the study setting (Primary Health Care), stakeholder groups (Patients), core focus (Smoking Cessation), conceptual construct (Health Knowledge, Attitudes, Practice), and geographical context (Iran). All selected keywords correspond to established MeSH terms to ensure appropriate indexing and discoverability.

Change Made:
The keywords have been updated to:
Primary Health Care; Dental Care; Patients; Smoking Cessation; Health Knowledge, Attitudes, Practice; Iran

Comment 12 (– Introduction):

Sources 1–6 are old and unreliable; very new sources are needed for epidemiology.

Response:

We appreciate this observation. The Introduction has been revised to reduce reliance on older references and to incorporate more recent epidemiological and clinical evidence. Where foundational references were retained (e.g., landmark or policy documents), they are now supplemented with more recent data sources to ensure the accuracy and contemporaneity of the evidence base. We have reduced reliance on older references in the opening paragraph and replaced or supplemented them with more recent studies.

Change Made:

- Replaced earlier epidemiological framing (global mortality + GDP burden) with **o**ral-health-focused, recent evidence:
    - Oral cancer, periodontitis, and implant failure now supported by newer or mixed-age sources.
- Introduced newer Iran-based and global studies
- Retained some older references only where they are foundational (e.g., Surgeon General), but no longer as sole support.

Comment 13 (– Introduction):

References are not in order in the first paragraph.

Response:

Thank you for pointing this out. The reference ordering throughout the Introduction has been carefully reviewed and corrected to ensure consistency with journal citation standards.

Change Made:

- All citations in the Introduction, including the first paragraph, have been reordered to follow proper sequential (Vancouver-style) numbering.

- The entire manuscript was rechecked to ensure consistent citation ordering after revisions.

Comment 14 (– Introduction):

The introduction lacks detailed and up-to-date background and reads more like epidemiology.

Response:

We appreciate this valuable feedback. The Introduction has been substantially restructured to move beyond descriptive epidemiology and provide a more analytical and conceptually grounded background. Specifically, we strengthened the linkage between epidemiological burden, clinical relevance, and intervention needs, and incorporated system-level and behavioral perspectives to contextualize the research problem.

Change Made:

- Reframed the opening to emphasize oral health consequences of tobacco use rather than general epidemiology alone.
- Added a dedicated paragraph outlining the importance of smoking cessation interventions and the role of primary care and dental settings.
- Introduced the concept of an implementation gap in SCCD delivery.
- Incorporated behavioral frameworks to explain the relevance of attitudes.

- Added discussion of dentist- and patient-level barriers to SCCD to provide a more comprehensive and analytical background.

Comment 15 (– Methods):

Confidentiality is mentioned twice; avoid repetition.

Response:
Thank you for this observation. We have revised the Procedure subsection to remove the repeated mention of confidentiality. The description has been streamlined to avoid redundancy while maintaining clarity regarding the informed consent process.

Change Made:
The second mention of confidentiality in the Research Procedure subsection has been removed. The informed consent description has been summarized to state that written informed consent was obtained as previously outlined.

Comment 16 (– Instrument):

Information about the instrument is incomplete. What is the scoring method?

Response:
Thank you for this helpful comment. In the previous version of the manuscript, although the Likert scale format, number of items, and total score ranges for the main constructs were reported, the scoring procedure may not have been sufficiently explicit for readers. In particular, the process by which individual item scores were summed to obtain total construct scores was not clearly stated.

To improve clarity, we have revised the Research Measures section to explicitly describe the scoring method used for each construct. The revised text now clearly indicates that each item was scored on a 5-point Likert scale (1 = strongly disagree to 5 = strongly agree) and that total construct scores were obtained by summing the responses across all items within each scale. We

also clarified the number of items included in each construct and subdomain and their corresponding score ranges**.**

Additionally, the descriptions of the patient and dentist attitude constructs were streamlined and reorganized to improve readability and avoid ambiguity regarding how the scores were calculated.

Change Made:
The Research Measures section (Dependent variables subsection) has been revised to:

- Explicitly state that construct scores were calculated by summing individual item scores**.**
- Clearly describe the Likert response scale (1–5) used for all attitude items.
- Specify the number of items included in each construct and domain.
- Report the resulting total score ranges for:
    - Overall patient SCCD attitudes (12–60)
    - Dentist SCCD competence (7–35)
    - SCCD effectiveness (3–15)
    - Oral symptoms as motivators (2–10)
    - Dentist SCCD attitudes (16–80)

These revisions clarify the scoring procedure and ensure that the measurement approach used in the study can be easily understood and replicated by readers.

Comment 17 (– Instrument):

Validity and reliability methods are unclear.

Response:
We appreciate this important observation. In the original version of the manuscript, we briefly cited the previously published study in which the questionnaire was developed and validated. Our intention was to avoid unnecessary repetition of the full validation procedures already documented in the original publication. However, we recognize that providing only a citation without summarizing the validation process may have limited the transparency of the instrument's psychometric properties for readers.

To address this issue, we have expanded the Methods section to summarize the key validation procedures conducted during the original development of the questionnaire, including the assessment of face validity by a panel of experts, content validity evaluation using structured ratings, and reliability testing through a test–retest procedure.

Change Made:
The "Research Tool" subsection has been substantially revised to include a concise summary of the questionnaire's original validation procedures, including face validity assessment, content validity evaluation, and reliability testing.

Comment 18 (– Instrument):

How was validity and reliability of the original questionnaire established?

Response:
Thank you for highlighting the need for greater clarity regarding the validation history of the questionnaire. In the original manuscript version, we referenced the study in which the questionnaire was developed and validated but did not summarize the specific validation procedures.

To improve transparency, the revised manuscript now briefly outlines the validation process reported in the original study. Specifically, face validity was assessed by a group of practicing dentists and an expert panel consisting of community oral health specialists and an epidemiologist. The panel further evaluated content validity by rating each item's relevance, simplicity, clarity, and necessity using a four-point scale, which resulted in a high scale content validity index. Reliability was assessed through a test–retest procedure with a two-week interval, producing item-level agreement coefficients ranging from 60% to 97.5%.

Change Made:
A description of the original validation procedures and reliability assessment has been added to the Research Tools subsection with appropriate citation to the source study.

Comment 19 (– Instrument):

The source is not reliable.

Response:
We appreciate the reviewer's concern regarding the credibility of the instrument source. The questionnaire used in the present study was adapted from a previously peer-reviewed study published in a Pubmed-indexed journal and conducted in comparable primary care dental settings. That study included a structured validation process involving expert review, content validity assessment, and test–retest reliability evaluation.

To address the reviewer's concern and enhance transparency, we have expanded the description of the original validation procedures in the Methods section. By summarizing the instrument development and psychometric evaluation conducted in the original study, we aim to clarify the methodological rigor underlying the questionnaire used in the present research.

Change Made:
The Methods section now includes a detailed summary of the validation procedures and reliability testing reported in the original study**,** with appropriate citation.

Comment 20 (– Methods Terminology):

Terms “dependent” and “independent variables” are more appropriate for experimental studies.

Response:
Thank you for this valuable comment. We have revised the terminology to better reflect the observational design of the study. The term “independent variables” has been replaced with “explanatory variables,” and “dependent variables” has been replaced with “outcome variables” throughout the manuscript

Change Made:
The term “independent variables” has been replaced with “explanatory variables,” and “dependent variables” has been replaced with “outcome variables” throughout the manuscript

Comment 21 (– Methods Clarity):

Tool description appears twice and is confusing.

Response:
Thank you for pointing out this issue. In the previous manuscript version, elements of the questionnaire description were presented in two separate parts of the Methods section, which may have created redundancy and reduced clarity.

In the revised manuscript, we have consolidated the description of the research instrument into a single, clearly structured subsection. This revision provides a more coherent explanation of the questionnaire’s structure, origin, scoring method, and validation history.

Change Made:
The **instrument description has been reorganized and consolidated** into a single subsection in the Methods section to improve clarity and eliminate redundancy.

Comment 22 (– Statistical Analyses):

Statistical analyses section should be more concise; descriptive information repeated in Table 1.

Response:
Thank you for this helpful suggestion. We have carefully revised the Statistical Analyses subsection to eliminate repetitive descriptive details that were already presented in Table 1. The section has been condensed to focus primarily on the analytical approach and modeling strategy, while avoiding redundancy with the descriptive results.

Change Made:
The Statistical Analyses subsection has been aggressively streamlined to reduce repetitive phrasing and over-detailed explanatory text while preserving full transparency regarding variable categorization, regression modeling strategy, and statistical thresholds.

Comment 23 (– Study Design Statement):

Based on findings, research method is descriptive cross-sectional; this was not mentioned.

Response:

Thank you for this thoughtful comment. The present study was designed as a cross-sectional study incorporating both descriptive and analytical components. The descriptive component provides a structured side-by-side comparison of SCCD attitudes among dentists and patients, while the analytical component examines associations between individual-level factors and attitude domains using regression modeling. We recognize that the prominence of the comparative descriptive presentation may have created ambiguity regarding the analytical aspect of the design. To clarify this, we have revised the Abstract, Introduction, Methods, Results, and Discussion sections to explicitly articulate both components of the study design and objectives.

Change Made:
The study design statement in the Abstract and Methods has been revised to clearly describe the study as a cross-sectional design incorporating both descriptive comparison and analytical modeling. The objective paragraph in the Introduction and the opening paragraph of the Discussion have been refined to distinguish between the comparative and determinant-focused aims. Additionally, clarifying transition statements have been added in the Results section to explicitly align descriptive analyses with the first objective and regression analyses with the second objective.

Comment 24 (– Discussion Structure):

Comparisons with literature are too detailed; background findings should be in introduction.

Response:

We appreciate this observation. In the previous version, the Discussion relied heavily on descriptive presentation of multiple studies per finding, often functioning as extended background summaries rather than analytical interpretation. In the revised version, we reduced citation density and reoriented the Discussion toward selective, determinant-based interpretation, where literature is used only to support or contrast specific findings . Background-style

explanations were removed, and literature is now embedded within analytical arguments rather than presented descriptively.

Changes made:

- Across all paragraphs:
  - Reduced number of cited studies to about 2 key references per finding
- Non-smoking determinant paragraph:
  - Consolidated literature across dentists and patients into one analytical block
  - Removed descriptive background explanations
- Employment determinant paragraph:
  - Reduced multiple supporting studies → retained key comparative and contrasting evidence
- Contextual determinant paragraphs:
  - Removed general system descriptions
  - Focused only on interpretation of findings
- Shift implemented across the manucript:
  - From: study-by-study description framing
  - To: finding → interpretation → targeted literature or finding → targeted literature → interpretation framing

Comment 25 (– References):

Most background sources are old and should be from the last 4 years.

Response:

We agree with the importance of using recent evidence and thank the reviewer for highlighting this point. The manuscript has been revised to incorporate a larger proportion of recent literature, particularly from the past 3–5 years. we have made a concerted effort to include more recent and relevant studies wherever possible, particularly when contextualizing findings and incorporating divergent evidence .

However, we note that the specific topic of stakeholder attitudes toward SCCD, especially from the patient perspective and within primary care dental settings, has received relatively limited and sporadic attention in the literature. As a result, the available evidence base remains constrained, and in several instances, the most directly comparable studies date back approximately a decade. Where more recent studies were not available, we retained these earlier sources due to their direct relevance to the constructs examined in this study. Importantly, these studies were used selectively and primarily to support or contrast specific findings, rather than to provide general background context. We have ensured that, wherever possible, older studies are complemented by more recent evidence in related domains, thereby maintaining both relevance and temporal balance in the interpretation.

Change Made:

- Increased the proportion of references from the last 4–5 years, including recent systematic reviews and primary studies.
- Added recent studies (2023–2026) for:
  - barriers to SCCD (e.g., Niven 2026, Chan 2023)
  - Iranian oral health and smoking outcomes
- Updated LMIC framing and primary care literature with newer sources (e.g., 2024 primary care cessation review).
- Updated key sections (epidemiology, SCCD effectiveness, and barriers) with contemporary sources.
- Reduced reliance on older references as primary support for key claims.

Across the Discussion:

- Prioritized inclusion of recent studies, particularly in:
  - employment/workplace influences
  - oral symptom-related motivation
  - SCCD implementation contexts

- Where older studies were retained:

  - Limited to cases where:
    - directly comparable evidence was unavailable
    - constructs closely matched study variables (e.g., patient SCCD attitudes)

- Integration strategy refined:

  - Older studies are:
    - used selectively
    - paired with newer evidence where feasible
    - embedded within analytical (not descriptive) interpretation

REVIEWER #2 COMMENTS

Comment 26 (– Abstract):

Verify terminology against MeSH for optimal indexing.

Response:
Thank you for this valuable suggestion. We have carefully reviewed the terminology used in the title, abstract, and keywords against the official Medical Subject Headings (MeSH) database to ensure optimal indexing and discoverability. The selected keywords have been revised to

correspond to established MeSH terms that accurately reflect the study's focus, setting, population, and conceptual framework.

Change Made:
The keywords have been revised and verified against the MeSH database. The final keyword list now includes the following MeSH terms:
Primary Health Care; Dental Care; Patients; Smoking Cessation; Health Knowledge, Attitudes, Practice; Iran.

Comment 27 (– Introduction):

Prevalence/incidence references outdated; update with current literature.

Response:

We thank the reviewer for this comment. The Introduction has been revised to include updated prevalence and burden estimates, particularly incorporating recent national and global data sources.

Change Made:

- Added:
    - STEPS 2021 national prevalence estimate (14%)
- Replaced earlier general prevalence statements with:
    - Iran-specific smoking and oral health data
- Added recent studies linking smoking to oral lesions and tissue repair in Iran.
- Revised prevalence-related statements to reflect current epidemiological understanding.

Comment 28 (– Introduction):

Multi-citation claims should reflect consensus.

Response:

We thank the reviewer for this clarification. We interpreted this comment as referring to the use of multiple citations to support single claims without clearly demonstrating that these studies collectively represent consistent evidence. In response, we revised the Introduction to ensure that grouped citations are used more selectively and only where they reflect converging findings across studies. Where appropriate, we reduced citation clustering and aligned each claim with the most directly relevant and representative sources.

Change Made:

- Reduced the use of long citation clusters (e.g., [23–26]) supporting single descriptive claims.

- Reorganized statements on dentist- and patient-related barriers to ensure that each citation directly supports the specific claim being made.
- Where multiple references are retained, ensured they represent consistent findings rather than loosely related evidence.
- Improved clarity by separating distinct concepts (e.g., dentist vs patient barriers) rather than aggregating them under shared citation groups.

Comment 29 (– Introduction):

Narrative lacks cohesiveness; research gap and rationale need clearer articulation.

Response:

Thank you for this insightful comment. The Introduction has been substantially restructured to improve narrative coherence and to clearly articulate the research gap and study rationale. The revised version follows a logical progression from problem identification to study justification.

Changes Made:

1. Reordered structure to:

    1. Oral health burden
    2. Need for cessation
    3. Delivery platforms (dentistry + primary care)
    4. Implementation gap
    5. Behavioral explanation
    6. Stakeholder barriers
    7. Research gap
    8. Study aim

2. Strengthened research gap using explicit statements:

"existing research has largely examined dentist and patient attitudes in isolation…"

And:

"few studies have explored multidimensional attitudinal constructs or individual-level determinants…"

3. Clarified study aim

Rewritten final paragraph:

- Explicit dual objectives:
    1. compare dentist vs patient attitudes
    2. examine determinants

- Explicit domains listed:
    - competence
    - effectiveness
    - oral symptoms

Comment 30 (– Methods):

Justify inclusion of participants aged 18 and above.

Response:
Thank you for this important clarification request. Participants aged 18 years and above were included because 18 is the legal age of adulthood in Iran, allowing individuals to provide independent informed consent without parental or guardian approval. Including minors would have introduced additional ethical obligations, including parental consent procedures and heightened protections for underage participants, which were beyond the scope of the present study design.

Furthermore, smoking behaviors, cessation decision-making, and oral health attitudes among adolescents differ substantially from those of adults and are shaped by distinct developmental, social, and behavioral determinants. Properly interpreting data from underage participants would therefore require a separate conceptual framework addressing adolescent oral health behavior and youth smoking patterns. Since the aim of this study was to examine attitudes toward smoking cessation counseling within routine adult primary care dental settings, restricting the sample to individuals aged 18 years and older ensured both ethical appropriateness and conceptual coherence with the study objectives.

Change Made:
A clarification has been added to the *Sample* subsection specifying that participants aged 18 years and above were included due to legal consent requirements in Iran and to maintain conceptual consistency with the study's focus on adult primary care dental patients.

Comment 31 (– Sample Clarification):

Clarify numbers 163, 188, and 367.

Response:
Thank you for this request for clarification. The number 163 refers to the minimum required sample size calculated for the dentist multiple linear regression analysis. The number 188 represents the total number of dentists working in CHCs in Tehran province at the time of the study. Given this relatively small and finite target population, a census approach was adopted to enhance representativeness, minimize sampling bias, and maximize statistical power by inviting all eligible dentists to participate.

For patients, the number 367 corresponds to the minimum required sample size estimated based on pilot data and predefined statistical parameters. To account for potential non-response and ensure adequate statistical power, slightly more participants were recruited, resulting in 380 completed questionnaires.

Change Made:
The Sample subsection has been revised to clearly distinguish between calculated minimum required sample sizes (163 for dentists and 367 for patients), the total eligible dentist population (188), and the final achieved patient sample (380). Additional explanatory text has been added to justify the census approach for dentists and the slight over-recruitment for patients.

Comment 32 (– Instrument Transparency):

Provide detailed development process, validation history, permissions, and psychometric properties.

Response:
We thank the reviewer for emphasizing the importance of instrument transparency. In the original manuscript, the questionnaire was described briefly and the original validation study was cited. Our intention was to avoid repeating methodological details already published in the original article. However, we acknowledge that additional summary information improves transparency and reader comprehension.

In response to this comment, we have expanded the description of the research instrument to include the key elements of its development and validation process. The revised text now summarizes the original face validity assessment conducted with practicing dentists and an expert panel, the structured evaluation of content validity using item ratings for relevance, simplicity, clarity, and necessity, and the reliability assessment performed through a two-week test–retest procedure.

Additionally, we clarify that selected items from this validated questionnaire were adapted for use in the present study and that the adapted instrument was piloted among a subset of dentists and patients to confirm clarity and contextual appropriateness

Change Made:
The **Research Tools subsection** has been substantially expanded to include a concise description of the questionnaire's development process, validation history, reliability assessment, and pilot testing in the present study.

Comment 33 (– Statistical Analysis):

Statistical section is excessively detailed and should be condensed.

Response:
Thank you for this helpful suggestion. We agree that the Statistical Analyses section contained

unnecessary detail and repetition. The subsection has been revised and condensed to improve clarity and readability while retaining essential methodological information.

Change Made:
The Statistical Analyses subsection has been aggressively streamlined to reduce repetitive phrasing and over-detailed explanatory text while preserving full transparency regarding variable categorization, regression modeling strategy, and statistical thresholds.

Comment 34 (– Results Tables):

Tables should be reviewed by a biostatistician and possibly merged.

Response:

Thank you for this constructive suggestion. In consultation with a biostatistician, we determined that merging Tables would reduce clarity, as these tables present results from distinct populations (dentists vs. patients) and different outcome measures (continuous attitude scores vs. dichotomized attitude domains vs. willingness to quit). Combining them would have made interpretation more difficult for readers.
Thus, to streamline the presentation while preserving the integrity of the data, we have:

- Moved Table 2 (bivariate associations for dentists) to a supplementary file (S1_Table), as this table primarily provides descriptive bivariate comparisons that support but are secondary to the main regression findings presented in the text and could have caused confusion in interpreting the results.
- Retained the other Tables the main manuscript, as they present infromation on the demographic makeup of the sample or results from regression analyses that address the study's primary analytical objectives.
- Updated the Results section to reflect this change, with a concise summary of the moved table's key findings and a clear reference to the supplementary file.
- Moved the text describing Table 2 to the section on predictors of dentist attitudes in a way that it precedes the presentation of predictors. This was done to more clearly depict the statistical pathway taken and avoid confusing the reader by spreading these findings all over the manucsript.

We believe these revisions improve the flow and focus of the main results while ensuring all data remain fully accessible to readers.

Comment 35 (– Discussion):

Discussion is overly lengthy; insufficient consideration of divergent evidence.

Response(A) Length:

We thank the reviewer for highlighting this issue. The Discussion has been condensed as much as possible through both structural and content-level revisions. In the previous version, length was driven by repetition and parallel presentation of similar findings across stakeholder groups .

In the revised version, organizing the Discussion around shared determinants across stakeholders enabled consolidation of overlapping content and removal of redundant explanations, resulting in a more concise and focused narrative **.**

Changes made:

- Merged overlapping dentist and patient discussions into unified determinant-based paragraphs:
    - smoking status
    - employment
- Removed duplicated conceptual blocks:
    - repeated dentist–patient contrast eliminated
- Condensed key paragraphs:
    - non-smoking
    - employment
    - contextual determinants
- Reduced citation density and eliminated redundancy across Discussion

---

(B) Divergent Evidence

Response: We appreciat the reviewer's point and have substantially strengthened engagement with divergent evidence. In the previous version, the Discussion largely presented confirmatory findings with limited critical comparison. In the revised version, divergent evidence is systematically incorporated within each determinant-focused paragraph, ensuring balanced and critical synthesis across all key findings . Each paragraph now includes supporting evidence, contrasting findings (where available), and explicit explanations for discrepancies based on methodological and contextual differences.

Changes made:

- For non-smoking, employment, and contextual determinants:
    - Added contradictory evidence
    - Explained potential reasons for the contradictions

Comment 36 (– Limitations):

Add a dedicated "Limitations" subsection.

Response:
Thank you for this helpful suggestion. We have added a dedicated "Strengths and Limitations" subsection to the Discussion section. This subsection explicitly outlines the methodological

limitations of the study. We have also clarified these factors should be considered when interpreting the findings.

Change Made:
A dedicated "Strengths and Limitations" subsection has been added to the Discussion section.

We again thank the Academic Editor and Reviewers for their valuable and constructive comments, which have substantially strengthened our manuscript. We hope that the revised version now meets the standards required for publication in PLOS ONE.

Sincerely,
Shabnam Varmazyari
(on behalf of all authors)
