# Supplementary material for: Smoking cessation counseling in primary care settings: Cross-sectional views from patients and dentists as key stakeholders
Source: PLoS One. 2026 Aug 14;21(8):e0354366. doi: 10.1371/journal.pone.0354366 (PMC13475981; doi:10.1371/journal.pone.0354366)
Supplement: Supplementary file 2 [file pone.0354366.s001.docx]

**Questionnaire for Assessing the Perspectives of Patients at Comprehensive Healthcare Centers Regarding Dental Smoking Cessation Counseling**

**Demographic Information**

**Date of Birth:**

**Gender:**

- 2-1. Female
- 2-2. Male

**Level of Education:**

- A1. Literate (able to read and write)
- A2. Primary school or middle school diploma
- A3. High school diploma or technical diploma
- A4. Associate degree
- A5. Bachelor's degree
- A6. Master's degree
- A7. Doctorate

**Occupation:**

- B1. Government employee
- B2. Government worker
- B3. Private sector employee
- B4. Private sector worker
- B5. Self-employed
- B6. Homemaker
- B7. Retired
- B8. Unemployed

**Main Questions**

1. Dentists should ask patients about tobacco use.
   1. Strongly Disagree
   2. Disagree
   3. Neutral
   4. Agree
   5. Strongly Agree
2. Dentists should explain the risks of tobacco use and its effects on oral and general health to tobacco users.
   1. Strongly Disagree
   2. Disagree
   3. Neutral
   4. Agree
   5. Strongly Agree
3. Dentists should assist smokers who are willing to quit.
   1. Strongly Disagree
   2. Disagree
   3. Neutral
   4. Agree
   5. Strongly Agree
4. Tobacco prevention should be part of routine oral health care and patient education.
   1. Strongly Disagree
   2. Disagree
   3. Neutral
   4. Agree
   5. Strongly Agree
5. Dental treatment is more important than tobacco cessation counseling.
   1. Strongly Disagree
   2. Disagree
   3. Neutral
   4. Agree
   5. Strongly Agree
6. A dentist should serve as a role model for patients and the general public and should not use tobacco.
   1. Strongly Disagree
   2. Disagree
   3. Neutral
   4. Agree
   5. Strongly Agree
7. Only physicians should provide tobacco cessation counseling to patients.
   1. Strongly Disagree
   2. Disagree
   3. Neutral
   4. Agree
   5. Strongly Agree
8. Most people do not quit tobacco use even if their dentist advises them to.
   1. Strongly Disagree
   2. Disagree
   3. Neutral
   4. Agree
   5. Strongly Agree
9. Talking to patients about quitting tobacco is pointless because most already know they should quit.
   1. Strongly Disagree
   2. Disagree
   3. Neutral
   4. Agree
   5. Strongly Agree
10. A patient's chances of quitting tobacco increase if they receive advice from a dentist.
    1. Strongly Disagree
    2. Disagree
    3. Neutral
    4. Agree
    5. Strongly Agree
11. Tooth discoloration caused by tobacco use is a sufficient motivation for quitting.
    1. Strongly Disagree
    2. Disagree
    3. Neutral
    4. Agree
    5. Strongly Agree
12. Bad breath caused by tobacco use is a sufficient motivation for quitting.
    1. Strongly Disagree
    2. Disagree
    3. Neutral
    4. Agree
    5. Strongly Agree

**Tobacco Use History**

13. **Do you smoke cigarettes?**

- 13-1. No, I have never smoked.
- 13-2. No, I used to smoke but have quit.
- 13-3. Yes, once a month or less.
- 13-4. Yes, two or three times a month.
- 13-5. Yes, two or three times a week.
- 13-6. Yes, once a day or more.

14. **Do you smoke a pipe?**

- 14-1. No, I have never smoked.
- 14-2. No, I used to smoke but have quit.
- 14-3. Yes, once a month or less.
- 14-4. Yes, two or three times a month.
- 14-5. Yes, two or three times a week.
- 14-6. Yes, once a day or more.

15. **Do you smoke hookah (waterpipe)?**

- 15-1. No, I have never smoked.
- 15-2. No, I used to smoke but have quit.
- 15-3. Yes, once a month or less.
- 15-4. Yes, two or three times a month.
- 15-5. Yes, two or three times a week.
- 15-6. Yes, once a day or more.

16. **Is there a smoker in your family? (Father, mother, spouse, sister, brother, child)**

- 16-1. Yes
- 16-2. No

17. **If you use any of the above tobacco products, would you like the dentist at the comprehensive healthcare center to help you quit?**

- 17-1. Yes
- 17-2. No
- 17-3. I do not currently use tobacco.
