# Supplementary material for: Smoking cessation counseling in primary care settings: Cross-sectional views from patients and dentists as key stakeholders
Source: PLoS One. 2026 Aug 14;21(8):e0354366. doi: 10.1371/journal.pone.0354366 (PMC13475981; doi:10.1371/journal.pone.0354366)
Supplement: Supplementary file 3 [file pone.0354366.s002.docx]

**Questionnaire for Assessing the Perspectives of Dentists at Comprehensive Healthcare Centers Regarding Dental Smoking Cessation Counseling**

**Date of birth:** ..............

**Gender:**
 - Female
 - Male

**Year of graduation from university:** ..............

**Employment status at the center:**
 - Government-mandated service (Tarh)
 - Permanent staff
 - Contractual
 - Other (please specify): __________

**Duration of service at the center:**
 - Less than 6 months
 - Less than 1 year
 - Less than 18 months
 - Less than 2 years
 - 2 to 5 years
 - 5 to 10 years
 - 10 to 15 years
 - 15 to 20 years
 - 20 to 25 years
 - 25 to 30 years

**Place of work (public system):**
 - Tehran city
 - Towns of Tehran province

**Do you smoke cigarettes?**
 - No, I have never smoked
 - No, I used to smoke but have quit
 - Yes, once a month or less
 - Yes, two or three times a month
 - Yes, two or three times a week
 - Yes, once a day or more

**Do you smoke a pipe?**
 - No, I have never smoked
 - No, I used to smoke but have quit
 - Yes, once a month or less
 - Yes, two or three times a month
 - Yes, two or three times a week
 - Yes, once a day or more

**Do you smoke hookah (waterpipe)?**
 - No, I have never smoked
 - No, I used to smoke but have quit
 - Yes, once a month or less
 - Yes, two or three times a month
 - Yes, two or three times a week
 - Yes, once a day or more

**Is there a smoker in your family?** (father, mother, spouse, sister, brother, child)
 - Yes
 - No

**Attitude items:**

1. Dentists should ask patients about tobacco smoking
     1- Strongly disagree 2- Disagree 3- Neutral 4- Agree 5- Strongly agree
2. Dentists should explain the risks of tobacco smoking and its effects on both oral and general health to tobacco users.
     1- Strongly disagree 2- Disagree 3- Neutral 4- Agree 5- Strongly agree
3. Dentists should help smokers who are willing to quit.
     1- Strongly disagree 2- Disagree 3- Neutral 4- Agree 5- Strongly agree
4. Tobacco smoking prevention should be a part of routine oral health education provided to patients.
     1- Strongly disagree 2- Disagree 3- Neutral 4- Agree 5- Strongly agree
5. Dentists are role models for their patients and the public and should not smoke tobacco.
     1- Strongly disagree 2- Disagree 3- Neutral 4- Agree 5- Strongly agree
6. Most people do not quit tobacco smoking even if their doctor advises them to.
     1- Strongly disagree 2- Disagree 3- Neutral 4- Agree 5- Strongly agree
7. It is useless to talk to patients about quitting tobacco because most of them already know they should quit.
     1- Strongly disagree 2- Disagree 3- Neutral 4- Agree 5- Strongly agree
8. If patients receive tobacco cessation counseling from their dentist, their chances of quitting increase.
     1- Strongly disagree 2- Disagree 3- Neutral 4- Agree 5- Strongly agree
9. I feel comfortable helping my patients quit tobacco smoking.
     1- Strongly disagree 2- Disagree 3- Neutral 4- Agree 5- Strongly agree
10. I am interested in providing tobacco cessation counseling to my patients.
      1- Strongly disagree 2- Disagree 3- Neutral 4- Agree 5- Strongly agree
11. I am interested in prescribing tobacco cessation medications to tobacco users who want to quit.
      1- Strongly disagree 2- Disagree 3- Neutral 4- Agree 5- Strongly agree
12. I am able to prescribe appropriate medications for tobacco cessation when needed.
      1- Strongly disagree 2- Disagree 3- Neutral 4- Agree 5- Strongly agree
13. I am able to provide tobacco cessation counseling to my patients according to guidelines.
      1- Strongly disagree 2- Disagree 3- Neutral 4- Agree 5- Strongly agree
14. I can support my smoking patients in quitting tobacco.
      1- Strongly disagree 2- Disagree 3- Neutral 4- Agree 5- Strongly agree
15. Dentists should receive specific training in tobacco cessation methods.
      1- Strongly disagree 2- Disagree 3- Neutral 4- Agree 5- Strongly agree
16. I feel the need to gain knowledge and skills to support patients in quitting tobacco smoking.
      1- Strongly disagree 2- Disagree 3- Neutral 4- Agree 5- Strongly agree
