# Supplementary material for: Smoking cessation counseling in primary care settings: Cross-sectional views from patients and dentists as key stakeholders
Source: PLoS One. 2026 Aug 14;21(8):e0354366. doi: 10.1371/journal.pone.0354366 (PMC13475981; doi:10.1371/journal.pone.0354366)
Supplement: Supplementary file 4 [file pone.0354366.s005.docx]

**Table 1. Comparison of SCCD**^†^ **attitudes across individual-level factors of primary care dentists in Tehran, Iran (n=173**^‡^**)**

| **P-value^§^** | **Mean Difference (95% Confidence Interval)** | **Mean dentist attitude score**  **(SD)** | **N** | **Variable category** | **Variable** |
| --- | --- | --- | --- | --- | --- |
| 0.515 | 1.2 (-2.4 to -4.9) | 56.3 (9.4) | 142 | Female^¶^ | Gender |
|  |  | 56.1 (9.6) | 32 | Male |  |
| 0.094 | -2.4 (-5.3 to -0.4) | 55.1 (9.3) | 122 | < 35^¶^ | Age |
|  |  | 57.5 (10.0) | 50 | ≥ 35 |  |
| 0.049* | -3.0 (-6.1 to -0.1) | 55.2 (9.38) | 112 | Under 5 years since graduation^¶^ | Education |
|  |  | 58.3 (9.48) | 56 | 5 years and above since graduation |  |
| 0.05 | -3.1 (-6.2 to 0.6) | 55.2 (9.22) | 125 | Governmental duty and contractual^¶^ | Occupation |
|  |  | 58.3 (9.71) | 49 | Official and others |  |
| 0.013* | 3.6 (0.8 to 6.3) | 58.0 (9.9) | 82 | City of Tehran^¶^ | Site of employment |
|  |  | 54.5 (8.7) | 91 | Bordering towns |  |
| 0.003* | -6.2 (-10.3 to -2.2) | 50.7 (8.2) | 23 | Yes^¶^ | Smoking status |
|  |  | 56.9 (9.3) | 151 | No |  |
| 0.400 | -1.4 (-4.6 to -1.8) | 55.2 (9.3) | 45 | Yes^¶^ | Smoking family member |
|  |  | 56.6 (9.4) | 127 | No |  |

^†^ Smoking cessation counseling by dentists

^‡^ Seven dentists (out of the 180 respondents) left at least one item in this section unanswered, leading to the exclusion of their responses from all analyses involved in the production of this table

^§^ Calculated using simple linear regression

^¶^ Reference category

^#*^Significant findings with p-values of less than 0.05.
