# Supplementary material for: Smoking cessation counseling in primary care settings: Cross-sectional views from patients and dentists as key stakeholders
Source: PLoS One. 2026 Aug 14;21(8):e0354366. doi: 10.1371/journal.pone.0354366 (PMC13475981; doi:10.1371/journal.pone.0354366)
Supplement: Supplementary file 5 [file pone.0354366.s006.docx]

**Abstract**

The method should specify the type of cross-sectional study: descriptive or analytical?

Keywords should be revised and adjusted to match the title. Many terms are not even in the abstract.

**Introduction**

Sources 1 to 6 are old and unreliable. Very new sources are needed for epidemiology.

In the first paragraph, the references are not in order.

The introduction lacks detailed and up-to-date background and is written more like an epidemiology.

**Method**

Confidentiality is mentioned once in the ethical principles and once again in the procedure, avoiding repetition of issues.

The information in the instrument is incomplete. What is the scoring method?

The validity and reliability method are not clear.

How is the validity and reliability of the original questionnaire?

The source is not reliable.

The terms dependent and independent variables belong to the experimental method. It is better to provide an alternative term.

Once in the tool section, the tool is given in general terms and once in the dependent variable. The reader is confused. These sections should be written more coherently.

Statistical analyses should be written more concisely. Descriptive information is not included in the introduction to this section and is repeated in Table 1.

Based on the findings, the research method is a descriptive cross-sectional study. This issue has not been mentioned anywhere.

Several comparisons with the research literature are written in detail in the discussion, which makes the reader unaware of the findings of the literature. These findings should come in introduction, and comparisons are discussed here. So, in this section, the comparisons will be summarized and the distinguishing feature of this study should be explained.

Most of the sources cited as research background are old and should be from the last 4 years.
