# Supplementary material for: Smoking cessation counseling in primary care settings: Cross-sectional views from patients and dentists as key stakeholders
Source: PLoS One. 2026 Aug 14;21(8):e0354366. doi: 10.1371/journal.pone.0354366 (PMC13475981; doi:10.1371/journal.pone.0354366)
Supplement: Supplementary file 6 [file pone.0354366.s008.docx]

**Dear Academic Editor and Reviewers,**

Thank you sincerely for your valuable feedback and the opportunity to revise our manuscript.

In accordance with the Academic Editor’s request to provide a new response letter addressing all previous and new comments in table format, we have organized this document into two parts.

Table 1 addresses the comments raised in the current revision round, including the Academic Editor’s formatting request and the brief current-round comments from Reviewer 1 and Reviewer 2. Tables 2–4 reproduce and itemize the comments and responses from the previous revision round. Specifically, Table 2 presents the previous Academic Editor / Journal comments, Table 3 presents the previous Reviewer 1 comments, and Table 4 presents the previous Reviewer 2 comments, with updated manuscript locations where applicable.

For each substantive manuscript revision, we indicate the corresponding manuscript page, paragraph or section, and line range based on the revised manuscript with line numbers. Where a change applies only to the response letter, cover letter, submission system, or supporting information files, this is stated explicitly rather than assigning an artificial manuscript location. Please keep in mind that even for the comments and responses pertaining to the previous evisio round, the change locations that have been provided belong to the most recent version of the manuscript that has been provided, given that provicding locations for the previous manusript version would simply be ineffective.

Of note is that the provided tracked changes version of the manuscript includes changes implemented **both during the current round of revision and the previous one.**

**Table 1** — Current revision-round comments

| **Source** | **Comment** | **Response and changes made** | **Location of changes in the manuscript** |
| --- | --- | --- | --- |
| Academic Editor / Comment to the author | Your response letter is currently difficult to follow and unsuitable for proper judgment. Please revise the format so that each reviewer comment is addressed separately, and for every response, clearly indicate the exact location (page and line number) of the corresponding changes in the revised manuscript. Use the following structure: Reviewer 1 / Comment 1 / Response 1: … (Page X, Line Y). Reviewer 2 / Comment 1 / Response 1: … (Page X, Line Y). Do not combine multiple comments or responses into one paragraph. Mark every change explicitly. Resubmit the response letter accordingly. Provide a new answer letter for all previous and new comments at the end of your manuscript. It is recommended to illustrate it in a table. | Thank you for this guidance. We have reformatted the response letter so that each reviewer/editor comment is addressed separately, with a distinct response and a corresponding location entry. Because the Academic Editor requested a new response letter covering all previous and new comments, the file is organized into an opening summary table followed by detailed sections. The opening summary table addresses the comments raised in the current revision round, including the formatting request and the brief current-round reviewer comments. The subsequent detailed sections reproduce and itemize the previous revision-round Academic Editor / Journal comments, Reviewer 1 comments, and Reviewer 2 comments, with updated manuscript locations where applicable. For changes made outside the manuscript body, we have explicitly stated that no manuscript page/line number is applicable. | Response-letter formatting change only; no manuscript-body location is applicable.  The manuscript-related locations for the substantive revisions are itemized in the detailed tables below. |
| Reviewer 1 Comment 1 | What is the reason for referencing in the setting section? | Thank you for this comment. The references in the Setting section were originally included to support contextual information necessary for understanding the study setting, particularly the structure and coverage of Iran’s primary healthcare system and the role of Comprehensive Health Centers (CHCs), where the study was conducted. Because these details may not be familiar to international readers, we retained the references directly supporting the healthcare-system context. However, we agree that unnecessary referencing should be avoided in the Setting section. Therefore, we revised the section to remove the demographic reference supporting Tehran province’s population status and limited the remaining references to essential contextual claims about Iran’s primary healthcare system and CHCs.  **Changes made:**  The Setting section was revised to make the use of references more focused and directly relevant to the study context. Specifically, the citation supporting general demographic information about Tehran province was removed, along with the phrase “status as Iran’s most populous province.” The wording describing CHCs was also refined by replacing “backbone” and “headquarters of the system” with the more precise phrase “central role.” The remaining references were retained only where they support essential information about Iran’s primary healthcare structure, CHC coverage, and CHC services. | Methods—Setting: Page 7, Lines 137–148 (revised CHC/primary-care-system context, Tehran-province rationale, and patient CHC selection). |
| Reviewer 1 Comment 2 | The validity and reliability of the questionnaires have not been stated. | Thank you for this comment. We expanded the Instrument and measures section to state the questionnaire source and summarize the relevant psychometric information. The revised text now explains that the attitude items were adapted from a previously validated questionnaire developed for primary care dental settings; face validity was assessed by dentists and an expert panel; content validity was assessed through structured ratings of relevance, simplicity, clarity, and necessity, yielding a scale content validity index of 0.96; reliability was evaluated using a two-week test–retest procedure with item-level agreement coefficients of 60%–97.5%; and the adapted instrument was piloted among 15 dentists and 15 patients in the present study.  In addition, in line with the note that the instrument had been previously validated in a similar setting, internal consistency for the adapted instruments was calculated using Cronbach’s alpha, with these results added to the Instrument and measures section. The dentist questionnaire demonstrated good internal consistency (α = 0.874). For the patient questionnaire, internal consistency was excellent for the SCCD effectiveness domain (α = 0.940) and acceptable for the oral symptoms as motivators domain (α = 0.729). However, the dentist SCCD competence domain showed limited internal consistency (α = 0.231). To address this transparently, a cautionary statement was added in the Instrument and measures section and this issue was further discussed in the Strengths and Limitations subsection. It was explained that this domain may not have captured a single homogeneous construct, possibly because its items reflect different aspects of perceived dentist role and competence. The items were retained to preserve content validity, but findings related to this domain are now explicitly interpreted cautiously. A future research recommendation was also added that this subscale should be further evaluated and refined before being used as a standalone measure.  **Change Made:** The Instrument and measures section was expanded to include the questionnaire source, original face/content validity procedures, scale CVI, test–retest reliability, pilot testing in the present study, and Cronbach’s alpha values for the adapted instruments and patient attitude domains. The Strengths and Limitations subsection was also revised to acknowledge the low internal consistency of the dentist SCCD competence domain, explain why the items were retained, and recommend future refinement of this subscale. | Methods—Instrument and measures: Page 9, Lines 180–198 and Page 10, Lines 199–204 (questionnaire source, original face/content validity, scale CVI, test–retest reliability, pilot testing, Cronbach’s alpha values, and cautious interpretation of the dentist SCCD competence domain). Discussion—Strengths and Limitations: Page 25, Lines 455–466 (low internal consistency limitation and rationale for cautious interpretation). Future studies: Page 26, Lines 479–484 (recommendation to further evaluate and refine the competence subscale). |
| Reviewer 1 Comment 3 | Instead of World Health Organization references, provide more accurate and up-to-date references. | Thank you for this comment.  We reviewed the WHO-related and older references throughout the manuscript and revised the reference list accordingly.  In the previous version, several broad background statements on tobacco-related oral health effects and tobacco cessation in oral health practice were supported mainly by older WHO documents, including the WHO monograph on tobacco cessation and oral health integration and the WHO fact sheet on tobacco and oral health.  In the revised manuscript, these broad evidentiary claims were replaced with more recent and more specific sources, including evidence on smoking-related oral/pharyngeal cancer, smoking-related periodontal outcomes, tobacco-related oral health consequences, dental implant failure, dental caries, and smoking-related oral lesions in Iranian populations.  We retained a WHO citation only where guideline-level support was specifically needed, replacing the older WHO source with the 2024 WHO clinical treatment guideline for tobacco cessation in adults. We also updated the oral-health-practitioner recommendation by citing recent FDI sources, including the 2022 FDI policy statement and the 2021 FDI tobacco cessation guidance for oral health professionals. In addition, the sentence on dentists’ role in cessation support was revised so that the rationale is now supported by recent and more specific sources. The revised text explains that dentists can reach large numbers of tobacco users through routine dental visits, identify tobacco-related oral manifestations, and use visible oral effects as motivation for quitting. | Introduction, paragraph 1: Page 4, Lines 66–76 (updated tobacco-related oral-health effects and Iran-specific oral-health/tobacco evidence). Introduction, paragraph 2: Page 4, Lines 77–86 and Page 5, Lines 87–88 (2024 WHO guideline, FDI recommendation, and dentists’ role/rationale for SCCD). References: Pages 28–32, Lines 523–628 (updated reference list, including recent WHO/FDI and oral-health/tobacco sources). |
| Reviewer 1 Comment 4 | Many of the sources are outdated. | Thank you for this important comment. We systematically reviewed the complete reference list and revised the manuscript to replace older, broad, redundant, or population-indirect citations with more recent and claim-specific evidence wherever suitable evidence was available. As a result, the reference list was reduced from 48 to 40 references: 18 references were removed, 10 recent or more directly relevant references were added, and 30 references were retained. The median publication year of the reference list increased from approximately 2018.5 to 2022, while the proportion of references published in 2020 or later increased from 47.9% to 72.5%. References published before 2015 were reduced from 11 to 3.  The most extensive revisions were made in the Introduction and Discussion. In the opening paragraph of the Introduction, broad WHO documents previously used to support multiple oral-health consequences of smoking were replaced with more recent and outcome-specific peer-reviewed evidence, including a recent meta-analysis of smoking and oral/pharyngeal cancer, a 2023 review of tobacco-related oral-health effects, a 2024 systematic review and meta-analysis of early dental implant failure, a systematic review of smoking and dental caries, and recent national evidence on periodontal disease in Iran. The previous WHO tobacco-cessation monograph was also replaced with the 2024 WHO clinical treatment guideline for tobacco cessation in adults, and current FDI policy and professional guidance were used for recommendations specific to oral-health practitioners.  We also removed older Iranian studies from 2011 and 2015 where the same points could be supported more directly by a 2024 study of Iranian primary-care dentists. In the Discussion, older studies concerning dentists’ implementation readiness and dental patients’ receptivity were replaced by more recent evidence published between 2021 and 2026. We additionally added recent evidence concerning dental-patient engagement and the relationship between providers’ smoking status, perceived barriers, perceived effectiveness, and confidence in cessation counselling. Several older and indirect interpretive references were removed, and the corresponding interpretations were narrowed or rewritten.  A limited number of older references were retained when they were foundational or remained the most direct source for a specific statement. For example, the original Theory of Planned Behavior publication was retained as a foundational theoretical source, while an older urban–rural dental-workforce study was retained because no sufficiently direct recent replacement was identified for that precise comparison. We have therefore prioritized relevance, methodological appropriateness, and claim specificity in addition to publication date.  These changes were made in the Introduction, including the paragraphs addressing tobacco-related oral-health effects, current cessation recommendations, SCCD implementation, and attitudinal barriers; in the Setting and Statistical Analyses subsections of the Methods; and throughout the Discussion paragraphs concerning implementation readiness, patient receptivity, smoking status, employment, family smoking, and urban–rural differences. The reference list and all in-text citation numbers were updated accordingly. | Introduction, paragraphs 1–4 and study aim: Page 4, Lines 66–86; Page 5, Lines 87–109; Page 6, Lines 110–118 (updated recent epidemiological, oral-health, SCCD, attitudinal-barrier, and rationale material). Methods—Setting and Statistical analyses: Page 7, Lines 137–148; Page 12, Lines 241–261; Page 13, Lines 262–265 (updated/retained contextual and analytical references where directly relevant). Discussion, determinant-based paragraphs: Page 20, Lines 348–375; Page 21, Lines 376–386; Page 22, Lines 387–409; Page 23, Lines 410–432; Page 24, Lines 433–448 (updated, narrowed, and balanced interpretive literature). References: Pages 28–32, Lines 523–628. |
| Reviewer 2 Comment 1 | No further comments left, I prefer the final decision be based on editor comments. Thanks for considering suggestion. | Thank you. We sincerely appreciate the reviewer’s note and have revised the manuscript and response letter in accordance with the Academic Editor’s comments and the remaining reviewer comments. | No manuscript-body change was requested by this comment. The substantive manuscript changes made in response to the Academic Editor and Reviewer 1 comments are itemized in the detailed sections below. |

**Table 2 —** Previous revision-round Academic Editor / Journal comments

| **Comment** | **Response and changes made** | **Location of changes in the manuscript** |
| --- | --- | --- |
| **Academic Editor Comment 1:**  The introduction must be updated with recent references to reflect the modern epidemiological landscape (not only from the last 4 years but broadly recent literature). | **Response:** We thank the Academic Editor for this important recommendation. The Introduction has been comprehensively revised to incorporate more recent epidemiological and contextual evidence. We prioritized the inclusion of up-to-date global and national data, particularly from recent WHO-aligned reports, national surveys, and contemporary peer-reviewed studies. Additionally, we strengthened the representation of Iran-specific evidence to better reflect the current epidemiological and health system context. We have also replaced several older foundational citations and supplementing retained ones with contemporary sources.  **Change Made:** • Replaced earlier global burden framing with updated distribution statement: • Added recent Iran-specific evidence:  o STEPS 2021 prevalence data  o Recent Iranian studies on oral mucosal lesions and caries among smokers (2024– 2025). • Updated oral health evidence base:  o Added a 2020 systematic review on smoking and periapical disease. • Shifted away from reliance on older WHO 2005-style epidemiology toward mixed recent sources. | Introduction, paragraph 1: Page 4, Lines 66–76. Introduction, paragraph 2: Page 4, Lines 77–86 and Page 5, Lines 87–88. Introduction, paragraph 3: Page 5, Lines 89–96. Introduction, paragraph 4: Page 5, Lines 97–109 and Page 6, Lines 110–112. Study aim: Page 6, Lines 113–118. References updated across Pages 28–32, Lines 523–628. |
| **Academic Editor Comment 2:**  Clarify the study design (e.g., descriptive vs analytical). | **Response:** We thank the Academic Editor for this important clarification. This comment overlaps with Reviewer comments regarding study design specification. In response, we have explicitly clarified throughout the manuscript that the study adopts a cross-sectional design incorporating both descriptive and analytical components. Especially, we have clarified the distinction between descriptive findings and analytical interpretation by beginning with the framing of desrcritpve findings around and then also restructuring the Discussion around shared analytical determinant domains across stakeholders. In the previous version, findings were often followed by descriptive summaries of literature. In the revised version, each paragraph begins with a specific finding and proceeds to analytical interpretation within a determinant-based framework, supported by selectively integrated literature.  **Change Made:** • Revised the Abstract to explicitly state: “This two-phase cross-sectional study, incorporating both descriptive and analytical elements…” • Revised the Study design and ethics subsection to clarify: “This cross-sectional study incorporated both descriptive comparison of stakeholder attitudes and analytical modeling…” • Refined the Introduction (final paragraph) to clearly distinguish between:  o the comparative (descriptive) objective, and  o the determinant-focused (analytical) objective. • Added transition language in the Results section linking descriptive analyses to the first objective and regression analyses to the second.  **Across the Discussion:**  • Restructured to begin with descriptive study findings and gaps followed by analytical findings (the indibidual-level determinants)  • Shift implemented from stakeholder-based narration to determinant-based analytical synthesis  • Added interpretive synthesis statements across paragraphs | Abstract—Materials and methods: Page 2, Lines 29–36. Introduction—study aim paragraph: Page 6, Lines 113–118. Methods—Study design and ethics paragraph: Page 6, Lines 123–131 and Page 7, Lines 132–136. Results transitions: Page 15, Lines 281–283 and Page 16, Lines 309–311. Discussion opening paragraph: Page 20, Lines 348–360. |
| **Academic Editor Comment 3:**  Provide clearer details regarding the validity, reliability, and psychometric properties of the research instruments. | **Response:** We appreciate this comment. This issue was also raised by Reviewer #1 (Comments 16–19) and Reviewer #2 (Comment 32). In response, we have expanded the description of the research instrument to provide a clear and transparent summary of its development, validation, and reliability assessment.  **Change Made:** • Expanded the “Instrument and measures” section to include:  o Face validity: assessed by 10 dentists and an expert panel (community oral health specialists and an epidemiologist), including evaluation of clarity and readability.  o Content validity: assessed via structured ratings (relevance, simplicity, clarity, necessity), resulting in a content validity index of 0.96.  o Reliability: described explicitly as a test–retest procedure with a two-week interval, with item-level agreement coefficients ranging from 60% to 97.5%. • Added clarification that the instrument was piloted in the present study (n=15 dentists, n=15 patients) to ensure contextual appropriateness and clarity. • Consolidated instrument description into a single, structured subsection to improve readability and avoid redundancy. | Methods—Instrument and measures: Page 9, Lines 180–198 and Page 10, Lines 199–204 (questionnaire source, face/content validity, CVI, reliability, pilot testing, Cronbach’s alpha values, and cautious interpretation of the low-internal-consistency competence domain). Explanatory/outcome variable description: Page 10, Lines 205–213. Scoring and attitude constructs: Page 10, Lines 214–219 and Page 11, Lines 220–239. Discussion—Strengths and Limitations: Page 25, Lines 455–466. Future studies: Page 26, Lines 479–484. |
| **Academic Editor Comment 4:**  Refine statistical reporting. | **Response:** We thank the Academic Editor for this suggestion. This comment overlaps with Reviewer #1 Comment 22 and Reviewer #2 Comment 33. In response, the Statistical Analyses section has been revised to improve clarity, reduce redundancy, and align reporting with standard conventions.  **Change Made (specific):** • Streamlined the Statistical analyses section by:  o Removing repeated descriptive explanations already presented in Table 1.  o Reorganizing variable categorization into a more concise and structured format. • Clarified analytical procedures by explicitly distinguishing:  o Descriptive analyses (means, SDs, frequencies)  o Comparative analyses (t-tests)  o Regression analyses (logistic and linear models) • Improved clarity of regression reporting by:  o Clearly defining outcome categorization thresholds  o Separating patient and dentist analytical strategies • Standardized terminology and improved readability by reducing overly detailed procedural language. | Methods—Statistical analyses: Page 12, Lines 241–261 and Page 13, Lines 262–265. These paragraphs contain the revised concise reporting of descriptive statistics, variable categorization, t-tests, logistic regression, linear regression, dichotomization thresholds, and significance criterion. |
| **Academic Editor Comment 5:** Streamline the discussion to provide a more cohesive and rigorous contribution. | **Response:** We thank the Academic Editor for this important comment. The Discussion has been fundamentally restructured to improve both cohesion and analytical rigor. In the previous version, the Discussion was organized in a largely segmented manner, with separate and partially  repetitive descriptions of dentist and patient perspectives, resulting in fragmentation and duplication. In the revised version, we reorganized the Discussion around shared determinant domains of SCCD attitudes across both stakeholder groups, including smoking status, employment, and contextual factors (practice setting for dentists and family smoking for patients). This restructuring enabled a transition from parallel descriptive narration to an integrated framework in which findings are interpreted mechanistically across stakeholders. Redundant contrasts between dentists and patients were removed, and each paragraph now addresses a single determinant within a unified explanatory narrative.  **Changes made:** • Global restructuring of Discussion:  o From: ▪ separate dentist and patient sections  o To: ▪ determinant-centered structure shared across stakeholders • Opening paragraph rewritten:  o Also an analytical framework (attitude–implementation gap + cross-stakeholder determinants) • Determinant-based paragraphs created:  o Non-smoking status  o Employment  o Context (practice setting + family smoking environment) • Redundant dentist–patient contrast removed from mid-Discussion | Discussion—opening and determinant-based synthesis: Page 20, Lines 348–375; Page 21, Lines 376–386; Page 22, Lines 387–409; Page 23, Lines 410–432; Page 24, Lines 433–448. Strengths/limitations and implications: Page 24, Lines 449–454; Page 25, Lines 455–478; Page 26, Lines 479–484. |
| **Journal Comment 6:**  Ensure manuscript meets PLOS ONE style requirements. | **Response:** Thank you for this reminder. We have carefully reviewed the revised manuscript to ensure full compliance with PLOS ONE formatting and style guidelines. This included standardizing section structure, reference formatting, in-text citation style, figure and table presentation, and terminology in accordance with journal requirements. We also performed a thorough editorial review of the manuscript to ensure consistency, clarity, and adherence to PLOS ONE submission standards. | Global formatting/style revision; not tied to a single manuscript location. Visible manuscript locations include Keywords: Page 3, Lines 49–50; Acknowledgements/AI-use statement: Page 27, Lines 501–506; Supporting information list: Page 33, Lines 632–638. |
| **Journal Comments 7–8:**  Funding information mismatch between “Funding Information” and “Financial Disclosure.” Clarify role of funders in study design, data collection, analysis, decision to publish, or manuscript preparation. | **Response:** Thank you for this query. As noted above, this study was supported through institutional funding from Tehran University of Medical Sciences (TUMS), for which no specific grant or award number is assigned. In this context, project identification and verification are linked to the study’s ethics approval. To ensure transparency, we have already provided the relevant ethics approval codes within the manuscript. In addition, the official ethics approval document—previously submitted as part of the supporting materials—clearly states the approval at the beginning of the document, followed by the corresponding ethics approval ID and detailed study specifications. These specifications (including study population characteristics) are consistent with those reported in the manuscript.  **Change Made:**  • Updated the Funding statement in the cover letter that read s:  “This study was supported in part by Tehran University of Medical Sciences (TUMS). The funder had no role in study design, data collection and analysis, decision to publish, or preparation of the manuscript.” | Submission system / cover letter change; not a manuscript-body text change. Relevant manuscript evidence for ethics approval appears in Methods—Study design and ethics: Page 6, Lines 123–131 and Page 7, Lines 132–136. |
| **Journal Comment 9:**  Remove or anonymize identifying data from Supporting Information files. | **Response:** Thank you for this important reminder. We have carefully reviewed all Supporting Information files and removed any potentially identifying information. All direct and indirect identifiers have been eliminated from both datasets. The revised Supporting Information files now include only variables that are directly relevant to the analyses and reported results presented in the manuscript.  **Change Made:** All Supporting Information datasets (S3 and S4 Files) have been revised to remove any potential identifiers. Only de-identified variables corresponding to the study analyses have been retained.  REVIEWER #1 COMMENTS | Supporting Information files S3 and S4; not a manuscript-body text change. The manuscript lists the de-identified supporting datasets in Supporting information: Page 33, Lines 632–638. |

**Table 3 —** Previous revision-round Reviewer 1 comments

| **Comment** | **Response and changes made** | Location of changes in the manuscript |
| --- | --- | --- |
| **Reviewer 1 Comment 1: (– Abstract):**  Specify whether the cross-sectional study is descriptive or analytical. | **Response:** Thank you for highlighting this point. The present study is a cross-sectional design incorporating both descriptive and analytical components. The descriptive component provides a structured comparison of SCCD attitudes between dentists and patients, while the analytical component examines associations between individual-level factors and attitude domains using regression modeling. To ensure clarity, we have revised the Abstract, Introduction, Methods, Results, and Discussion sections to explicitly specify both components of the study design and objectives.  **Change Made:**  • The study design statement in the Abstract and Methods sections now clearly indicates that the study combines descriptive comparison with analytical modeling.  • The Introduction and Discussion opening paragraphs have been refined to differentiate the comparative descriptive component from the analytical examination of determinants. | Abstract—Materials and methods: Page 2, Lines 29–36. Introduction—study aim paragraph: Page 6, Lines 113–118. Methods—Study design and ethics: Page 6, Lines 123–131 and Page 7, Lines 132–136. Results transitions: Page 15, Lines 281–283 and Page 16, Lines 309–311. Discussion opening: Page 20, Lines 348–360. |
| **Reviewer 1 Comment 2: (– Abstract):**  Keywords should be revised to match the title and abstract. | **Response:** Thank you for this helpful suggestion. The keywords have been revised to better align with the terminology and core constructs emphasized in the title and abstract. Specifically, we ensured that the selected terms reflect the study setting (Primary Health Care), stakeholder groups (Patients), core focus (Smoking Cessation), conceptual construct (Health Knowledge, Attitudes, Practice), and geographical context (Iran). All selected keywords correspond to established MeSH terms to ensure appropriate indexing and discoverability.  **Change Made:** The keywords have been updated to: Primary Health Care; Dental Care; Patients; Smoking Cessation; Health Knowledge, Attitudes, Practice; Iran | Keywords: Page 3, Lines 49–50. |
| **Reviewer 1 Comment 3: (– Introduction):**  Sources 1–6 are old and unreliable; very new sources are needed for epidemiology. | **Response:**  We appreciate this observation. The Introduction has been revised to reduce reliance on older references and to incorporate more recent epidemiological and clinical evidence. Where foundational references were retained (e.g., landmark or policy documents), they are now supplemented with more recent data sources to ensure the accuracy and contemporaneity of the evidence base. We have reduced reliance on older references in the opening paragraph and replaced or supplemented them with more recent studies.  **Change Made:** • Replaced earlier epidemiological framing (global mortality + GDP burden) with oral- health-focused, recent evidence:  o Oral cancer, periodontitis, and implant failure now supported by newer or mixed- age sources. • Introduced newer Iran-based and global studies • Retained some older references only where they are foundational (e.g., Surgeon General), but no longer as sole support. | Introduction, paragraph 1: Page 4, Lines 66–76. Introduction, paragraph 2: Page 4, Lines 77–86 and Page 5, Lines 87–88. Recent/updated references appear throughout the reference list, especially Pages 28–32, Lines 523–628. |
| **Reviewer 1 Comment 4: (– Introduction):**  References are not in order in the first paragraph. | **Response:** Thank you for pointing this out. The reference ordering throughout the Introduction has been carefully reviewed and corrected to ensure consistency with journal citation standards.  **Change Made:**  • All citations in the Introduction, including the first paragraph, have been reordered to follow proper sequential (Vancouver-style) numbering.  • The entire manuscript was rechecked to ensure consistent citation ordering after revisions. | Introduction, paragraph 1: Page 4, Lines 66–76. This paragraph contains the reordered first-paragraph citations. |
| **Reviewer 1 Comment 5: (– Introduction):**  The introduction lacks detailed and up-to-date background and reads more like epidemiology. | **Response:** We appreciate this valuable feedback. The Introduction has been substantially restructured to move beyond descriptive epidemiology and provide a more analytical and conceptually grounded background. Specifically, we strengthened the linkage between epidemiological burden, clinical relevance, and intervention needs, and incorporated system-level and behavioral perspectives to contextualize the research problem.  **Change Made:**  • Reframed the opening to emphasize oral health consequences of tobacco use rather than general epidemiology alone.  • Added a dedicated paragraph outlining the importance of smoking cessation interventions and the role of primary care and dental settings.  • Introduced the concept of an implementation gap in SCCD delivery.  • Incorporated behavioral frameworks to explain the relevance of attitudes.  • Added discussion of dentist- and patient-level barriers to SCCD to provide a more comprehensive and analytical background. | Introduction, paragraph 1: Page 4, Lines 66–76. Introduction, paragraph 2: Page 4, Lines 77–86 and Page 5, Lines 87–88. Introduction, paragraph 3: Page 5, Lines 89–96. Introduction, paragraph 4: Page 5, Lines 97–109 and Page 6, Lines 110–112. Study aim: Page 6, Lines 113–118. |
| **Reviewer 1 Comment 6: (– Methods):**  Confidentiality is mentioned twice; avoid repetition. | **Response:** Thank you for this observation. We have revised the Procedure subsection to remove the repeated mention of confidentiality. The description has been streamlined to avoid redundancy while maintaining clarity regarding the informed consent process.  **Change Made:** The second mention of confidentiality in the Research Procedure subsection has been removed. The informed consent description has been summarized to state that written informed consent was obtained as previously outlined. | Methods—Study design and ethics, consent/confidentiality paragraph: Page 6, Lines 123–131 and Page 7, Lines 132–136. Methods—Procedure, streamlined consent reference: Page 8, Lines 174–176 and Page 9, Lines 177–179. |
| **Reviewer 1 Comment 7: (– Instrument):**  Information about the instrument is incomplete. What is the scoring method? | **Response:** Thank you for this helpful comment. In the previous version of the manuscript, although the Likert scale format, number of items, and total score ranges for the main constructs were reported, the scoring procedure may not have been sufficiently explicit for readers. In particular, the process by which individual item scores were summed to obtain total construct scores was not clearly stated. To improve clarity, we have revised the Research Measures section to explicitly describe the scoring method used for each construct. The revised text now clearly indicates that each item was scored on a 5-point Likert scale (1 = strongly disagree to 5 = strongly agree) and that total construct scores were obtained by summing the responses across all items within each scale. We  also clarified the number of items included in each construct and subdomain and their corresponding score ranges. Additionally, the descriptions of the patient and dentist attitude constructs were streamlined and reorganized to improve readability and avoid ambiguity regarding how the scores were calculated.  **Change Made:** The Research Measures section (Dependent variables subsection) has been revised to: • Explicitly state that construct scores were calculated by summing individual item scores. • Clearly describe the Likert response scale (1–5) used for all attitude items. • Specify the number of items included in each construct and domain. • Report the resulting total score ranges for:  o Overall patient SCCD attitudes (12–60)  o Dentist SCCD competence (7–35)  o SCCD effectiveness (3–15)  o Oral symptoms as motivators (2–10)  o Dentist SCCD attitudes (16–80) These revisions clarify the scoring procedure and ensure that the measurement approach used in the study can be easily understood and replicated by readers. | Methods—Instrument and measures, scoring of patient attitude domains: Page 10, Lines 214–219 and Page 11, Lines 220–232. Dentist SCCD attitude scoring: Page 11, Lines 233–239. |
| **Reviewer 1 Comment 8: (– Instrument):**  Validity and reliability methods are unclear. | **Response:** We appreciate this important observation. In the original version of the manuscript, we briefly cited the previously published study in which the questionnaire was developed and validated. Our intention was to avoid unnecessary repetition of the full validation procedures already documented in the original publication. However, we recognize that providing only a citation without summarizing the validation process may have limited the transparency of the instrument’s psychometric properties for readers. To address this issue, we have expanded the Methods section to summarize the key validation procedures conducted during the original development of the questionnaire, including the assessment of face validity by a panel of experts, content validity evaluation using structured ratings, and reliability testing through a test–retest procedure.  **Change Made:** The “Research Tool” subsection has been substantially revised to include a concise summary of the questionnaire’s original validation procedures, including face validity assessment, content validity evaluation, and reliability testing. | Methods—Instrument and measures, validation/reliability summary: Page 9, Lines 180–198 and Page 10, Lines 199–204. |
| **Reviewer 1 Comment 9: (– Instrument):**  How was validity and reliability of the original questionnaire established? | **Response:** Thank you for highlighting the need for greater clarity regarding the validation history of the questionnaire. In the original manuscript version, we referenced the study in which the questionnaire was developed and validated but did not summarize the specific validation procedures. To improve transparency, the revised manuscript now briefly outlines the validation process reported in the original study. Specifically, face validity was assessed by a group of practicing dentists and an expert panel consisting of community oral health specialists and an epidemiologist. The panel further evaluated content validity by rating each item’s relevance, simplicity, clarity, and necessity using a four-point scale, which resulted in a high scale content validity index. Reliability was assessed through a test–retest procedure with a two-week interval, producing item-level agreement coefficients ranging from 60% to 97.5%.  **Change Made:** A description of the original validation procedures and reliability assessment has been added to the Research Tools subsection with appropriate citation to the source study. | Methods—Instrument and measures, original validation/reliability procedures: Page 9, Lines 185–193. Present-study pilot testing and internal-consistency information: Page 9, Lines 194–198 and Page 10, Lines 199–204. |
| **Reviewer 1 Comment 10: (– Instrument):**  The source is not reliable. | **Response:** We appreciate the reviewer’s concern regarding the credibility of the instrument source. The questionnaire used in the present study was adapted from a previously peer-reviewed study published in a Pubmed-indexed journal and conducted in comparable primary care dental settings. That study included a structured validation process involving expert review, content validity assessment, and test–retest reliability evaluation. To address the reviewer’s concern and enhance transparency, we have expanded the description of the original validation procedures in the Methods section. By summarizing the instrument development and psychometric evaluation conducted in the original study, we aim to clarify the methodological rigor underlying the questionnaire used in the present research.  **Change Made:** The Methods section now includes a detailed summary of the validation procedures and reliability testing reported in the original study, with appropriate citation. | Methods—Instrument and measures, questionnaire source and validation/reliability history: Page 9, Lines 181–193. Present-study pilot testing and internal-consistency information: Page 9, Lines 194–198 and Page 10, Lines 199–204. |
| **Reviewer 1 Comment 11: (– Methods Terminology):**  Terms “dependent” and “independent variables” are more appropriate for experimental studies. | **Response:** Thank you for this valuable comment. We have revised the terminology to better reflect the observational design of the study. The term “independent variables” has been replaced with  “explanatory variables,” and “dependent variables” has been replaced with “outcome variables” throughout the manuscript  **Change Made:** The term “independent variables” has been replaced with “explanatory variables,” and  “dependent variables” has been replaced with “outcome variables” throughout the manuscript | Methods—Instrument and measures, explanatory variables and outcome variables: Page 10, Lines 205–213. Methods—Sample and Statistical analyses, observational terminology in modeling context: Page 8, Lines 164–173; Page 12, Lines 244–261; Page 13, Lines 262–265. |
| **Reviewer 1 Comment 12: (– Methods):**  Tool description appears twice and is confusing.  **.** | **Response:** Thank you for pointing out this issue. In the previous manuscript version, elements of the questionnaire description were presented in two separate parts of the Methods section, which may have created redundancy and reduced clarity. In the revised manuscript, we have consolidated the description of the research instrument into a single, clearly structured subsection. This revision provides a more coherent explanation of the questionnaire’s structure, origin, scoring method, and validation history.  **Change Made:** The instrument description has been reorganized and consolidated into a single subsection in the Methods section to improve clarity and eliminate redundancy. | Methods—Instrument and measures, consolidated subsection: Page 9, Lines 180–198; Page 10, Lines 199–219; Page 11, Lines 220–239. |
| **Reviewer 1 Comment 13: (– Statistical Analyses):**  Statistical analyses section should be more concise; descriptive information repeated in Table 1. | **Response:** Thank you for this helpful suggestion. We have carefully revised the Statistical Analyses subsection to eliminate repetitive descriptive details that were already presented in Table 1. The section has been condensed to focus primarily on the analytical approach and modeling strategy, while avoiding redundancy with the descriptive results.  **Change Made:** The Statistical Analyses subsection has been aggressively streamlined to reduce repetitive phrasing and over-detailed explanatory text while preserving full transparency regarding variable categorization, regression modeling strategy, and statistical thresholds. | Methods—Statistical analyses: Page 12, Lines 241–261 and Page 13, Lines 262–265. |
| **Reviewer 1 Comment 14: (– Study Design Statement):**  Based on findings, research method is descriptive cross-sectional; this was not mentioned. | **Response:** Thank you for this thoughtful comment. The present study was designed as a cross-sectional study incorporating both descriptive and analytical components. The descriptive component provides a structured side-by-side comparison of SCCD attitudes among dentists and patients, while the analytical component examines associations between individual-level factors and attitude domains using regression modeling. We recognize that the prominence of the comparative descriptive presentation may have created ambiguity regarding the analytical aspect of the design. To clarify this, we have revised the Abstract, Introduction, Methods, Results, and Discussion sections to explicitly articulate both components of the study design and objectives.  **Change Made:** The study design statement in the Abstract and Methods has been revised to clearly describe the study as a cross-sectional design incorporating both descriptive comparison and analytical modeling. The objective paragraph in the Introduction and the opening paragraph of the Discussion have been refined to distinguish between the comparative and determinant-focused aims. Additionally, clarifying transition statements have been added in the Results section to explicitly align descriptive analyses with the first objective and regression analyses with the second objective. | Abstract—Materials and methods: Page 2, Lines 29–36. Introduction—study aim paragraph: Page 6, Lines 113–118. Methods—Study design and ethics: Page 6, Lines 123–131 and Page 7, Lines 132–136. Results transitions: Page 15, Lines 281–283 and Page 16, Lines 309–311. |
| **Reviewer 1 Comment 15: (– Discussion Structure):**  Comparisons with literature are too detailed; background findings should be in introduction. | **Response:** We appreciate this observation. In the previous version, the Discussion relied heavily on descriptive presentation of multiple studies per finding, often functioning as extended background summaries rather than analytical interpretation. In the revised version, we reduced citation density and reoriented the Discussion toward selective, determinant-based interpretation, where literature is used only to support or contrast specific findings. Background-style  explanations were removed, and literature is now embedded within analytical arguments rather than presented descriptively.  **Changes made:** • Across all paragraphs:  o Reduced number of cited studies to about 2 key references per finding • Non-smoking determinant paragraph:  o Consolidated literature across dentists and patients into one analytical block  o Removed descriptive background explanations • Employment determinant paragraph:  o Reduced multiple supporting studies → retained key comparative and contrasting evidence • Contextual determinant paragraphs:  o Removed general system descriptions  o Focused only on interpretation of findings • Shift implemented across the manucript:  o From: study-by-study description framing  o To: finding → interpretation → targeted literature or finding → targeted literature → interpretation framing | Background strengthened in Introduction: Page 4, Lines 66–86; Page 5, Lines 87–109; Page 6, Lines 110–118. Streamlined Discussion: Page 20, Lines 348–375; Page 21, Lines 376–386; Page 22, Lines 387–409; Page 23, Lines 410–432; Page 24, Lines 433–448. |
| **Reviewer 1 Comment 16: (– References):**  Most background sources are old and should be from the last 4 years. | **Response:** We agree with the importance of using recent evidence and thank the reviewer for highlighting this point. The manuscript has been revised to incorporate a larger proportion of recent literature, particularly from the past 3–5 years. we have made a concerted effort to include more recent and relevant studies wherever possible, particularly when contextualizing findings and incorporating divergent evidence. However, we note that the specific topic of stakeholder attitudes toward SCCD, especially from the patient perspective and within primary care dental settings, has received relatively limited and sporadic attention in the literature. As a result, the available evidence base remains constrained, and in several instances, the most directly comparable studies date back approximately a decade. Where more recent studies were not available, we retained these earlier sources due to their direct relevance to the constructs examined in this study. Importantly, these studies were used selectively and primarily to support or contrast specific findings, rather than to provide general background context. We have ensured that, wherever possible, older studies are complemented by more recent evidence in related domains, thereby maintaining both relevance and temporal balance in the interpretation.  **Change Made:** • Increased the proportion of references from the last 4–5 years, including recent systematic reviews and primary studies. • Added recent studies (2023–2026) for:  o barriers to SCCD (e.g., Niven 2026, Chan 2023)  o Iranian oral health and smoking outcomes • Updated LMIC framing and primary care literature with newer sources (e.g., 2024 primary care cessation review). • Updated key sections (epidemiology, SCCD effectiveness, and barriers) with contemporary sources. • Reduced reliance on older references as primary support for key claims.  **Across the Discussion:** • Prioritized inclusion of recent studies, particularly in:  o employment/workplace influences  o oral symptom-related motivation  o SCCD implementation contexts  • Where older studies were retained: • Limited to cases where:  o directly comparable evidence was unavailable  o constructs closely matched study variables (e.g., patient SCCD attitudes)  • Integration strategy refined: • Older studies are:  o used selectively  o paired with newer evidence where feasible  o embedded within analytical (not descriptive) interpretation  REVIEWER #2 COMMENTS | Introduction and rationale updated with recent sources: Page 4, Lines 66–86; Page 5, Lines 87–109; Page 6, Lines 110–118. Discussion updated and narrowed: Page 20, Lines 348–375; Page 21, Lines 376–386; Page 22, Lines 387–409; Page 23, Lines 410–432; Page 24, Lines 433–448. Updated reference list: Pages 28–32, Lines 523–628. |

**Table 4 —** Previous revision-round Reviewer 2 comments

| **Comment** | **Response and changes made** | Location of changes in the manuscript |
| --- | --- | --- |
| **Reviewer 2 Comment 1: (– Abstract):**  Verify terminology against MeSH for optimal indexing. | **Response:** Thank you for this valuable suggestion. We have carefully reviewed the terminology used in the title, abstract, and keywords against the official Medical Subject Headings (MeSH) database to ensure optimal indexing and discoverability. The selected keywords have been revised to  correspond to established MeSH terms that accurately reflect the study’s focus, setting, population, and conceptual framework.  **Change Made:** The keywords have been revised and verified against the MeSH database. The final keyword list now includes the following MeSH terms: Primary Health Care; Dental Care; Patients; Smoking Cessation; Health Knowledge, Attitudes, Practice; Iran. | Keywords: Page 3, Lines 49–50. |
| **Reviewer 2 Comment 2: (– Introduction):**  Prevalence/incidence references outdated; update with current literature. | **Response:** We thank the reviewer for this comment. The Introduction has been revised to include updated prevalence and burden estimates, particularly incorporating recent national and global data sources.  **Change Made:** • Added:  o STEPS 2021 national prevalence estimate (14%) • Replaced earlier general prevalence statements with:  o Iran-specific smoking and oral health data • Added recent studies linking smoking to oral lesions and tissue repair in Iran. • Revised prevalence-related statements to reflect current epidemiological understanding. | Introduction, paragraph 1: Page 4, Lines 66–76; Introduction, paragraph 2: Page 4, Lines 77–86 and Page 5, Lines 87–88. Updated epidemiology/Iran-specific references appear in References: Page 28, Lines 523–541 and Page 29, Lines 542–563. |
| **Reviewer 2 Comment 3: (– Introduction):**  Multi-citation claims should reflect consensus. | **Response:** We thank the reviewer for this clarification. We interpreted this comment as referring to the use of multiple citations to support single claims without clearly demonstrating that these studies collectively represent consistent evidence. In response, we revised the Introduction to ensure that grouped citations are used more selectively and only where they reflect converging findings across studies. Where appropriate, we reduced citation clustering and aligned each claim with the most directly relevant and representative sources.  **Change Made:** • Reduced the use of long citation clusters (e.g., [23–26]) supporting single descriptive claims.  • Reorganized statements on dentist- and patient-related barriers to ensure that each citation directly supports the specific claim being made. • Where multiple references are retained, ensured they represent consistent findings rather than loosely related evidence. • Improved clarity by separating distinct concepts (e.g., dentist vs patient barriers) rather than aggregating them under shared citation groups. | Introduction, implementation gap and attitudinal-barrier paragraphs: Page 5, Lines 89–109 and Page 6, Lines 110–112. These lines separate implementation-gap, behavioral-framework, dentist-barrier, patient-barrier, and research-gap claims. |
| **Reviewer 2 Comment 4: (– Introduction):**  Narrative lacks cohesiveness; research gap and rationale need clearer articulation. | **Response:** Thank you for this insightful comment. The Introduction has been substantially restructured to improve narrative coherence and to clearly articulate the research gap and study rationale. The revised version follows a logical progression from problem identification to study justification.  **Changes Made:**  1. Reordered structure to:  1. Oral health burden  2. Need for cessation  3. Delivery platforms (dentistry + primary care)  4. Implementation gap  5. Behavioral explanation  6. Stakeholder barriers  7. Research gap  8. Study aim  2. Strengthened research gap using explicit statements:  “existing research has largely examined dentist and patient attitudes in isolation…”  And:  “few studies have explored multidimensional attitudinal constructs or individual-level determinants…”  3. Clarified study aim Rewritten final paragraph: • Explicit dual objectives:  1. compare dentist vs patient attitudes  2. examine determinants  • Explicit domains listed:  o competence  o effectiveness  o oral symptoms | Introduction, full revised narrative sequence: Page 4, Lines 66–86; Page 5, Lines 87–109; Page 6, Lines 110–118. |
| **Reviewer 2 Comment 5: (– Methods):**  Justify inclusion of participants aged 18 and above. | **Response:** Thank you for this important clarification request. Participants aged 18 years and above were included because 18 is the legal age of adulthood in Iran, allowing individuals to provide independent informed consent without parental or guardian approval. Including minors would have introduced additional ethical obligations, including parental consent procedures and heightened protections for underage participants, which were beyond the scope of the present study design. Furthermore, smoking behaviors, cessation decision-making, and oral health attitudes among adolescents differ substantially from those of adults and are shaped by distinct developmental, social, and behavioral determinants. Properly interpreting data from underage participants would therefore require a separate conceptual framework addressing adolescent oral health behavior and youth smoking patterns. Since the aim of this study was to examine attitudes toward smoking cessation counseling within routine adult primary care dental settings, restricting the sample to individuals aged 18 years and older ensured both ethical appropriateness and conceptual coherence with the study objectives.  **Change Made:** A clarification has been added to the Sample subsection specifying that participants aged 18 years and above were included due to legal consent requirements in Iran and to maintain conceptual consistency with the study’s focus on adult primary care dental patients. | Methods—Sample, adult eligibility rationale: Page 7, Lines 149–153 and Page 8, Lines 154–157. |
| **Reviewer 2 Comment 6: (– Sample Clarification):**  Clarify numbers 163, 188, and 367. | **Response:** Thank you for this request for clarification. The number 163 refers to the minimum required sample size calculated for the dentist multiple linear regression analysis. The number 188 represents the total number of dentists working in CHCs in Tehran province at the time of the study. Given this relatively small and finite target population, a census approach was adopted to enhance representativeness, minimize sampling bias, and maximize statistical power by inviting all eligible dentists to participate.  For patients, the number 367 corresponds to the minimum required sample size estimated based on pilot data and predefined statistical parameters. To account for potential non-response and ensure adequate statistical power, slightly more participants were recruited, resulting in 380 completed questionnaires.  **Change Made:** The Sample subsection has been revised to clearly distinguish between calculated minimum required sample sizes (163 for dentists and 367 for patients), the total eligible dentist population (188), and the final achieved patient sample (380). Additional explanatory text has been added to justify the census approach for dentists and the slight over-recruitment for patients. | Methods—Sample, patient and dentist sample-size clarification: Page 8, Lines 158–173. |
| **Reviewer 2 Comment 7: (– Instrument Transparency):**  Provide detailed development process, validation history, permissions, and psychometric properties. | **Response:** We thank the reviewer for emphasizing the importance of instrument transparency. In the original manuscript, the questionnaire was described briefly and the original validation study was cited. Our intention was to avoid repeating methodological details already published in the original article. However, we acknowledge that additional summary information improves transparency and reader comprehension. In response to this comment, we have expanded the description of the research instrument to include the key elements of its development and validation process. The revised text now summarizes the original face validity assessment conducted with practicing dentists and an expert panel, the structured evaluation of content validity using item ratings for relevance, simplicity, clarity, and necessity, and the reliability assessment performed through a two-week test–retest procedure. Additionally, we clarify that selected items from this validated questionnaire were adapted for use in the present study and that the adapted instrument was piloted among a subset of dentists and patients to confirm clarity and contextual appropriateness.  In addition, if by permission, permission from the research team reponsible for the development of the very initial questionaire of the prior study is being referred to, it was not necessary as it was basically the same research team invovled in the current study we are addressing in this review.  **Change Made:** The Research Tools subsection has been substantially expanded to include a concise description of the questionnaire’s development process, validation history, reliability assessment, and pilot testing in the present study. | Methods—Instrument and measures: Page 9, Lines 180–198 and Page 10, Lines 199–204 (source, validation history, reliability, pilot testing, internal consistency, and cautionary interpretation). Variables/domains/scoring and outcome construction: Page 10, Lines 205–219 and Page 11, Lines 220–239. Discussion—Strengths and Limitations: Page 25, Lines 455–466. Future studies: Page 26, Lines 479–484. |
| **Reviewer 2 Comment 8: (– Statistical Analysis):**  Statistical section is excessively detailed and should be condensed. | **Response:** Thank you for this helpful suggestion. We agree that the Statistical Analyses section contained  unnecessary detail and repetition. The subsection has been revised and condensed to improve clarity and readability while retaining essential methodological information.  **Change Made:** The Statistical Analyses subsection has been aggressively streamlined to reduce repetitive phrasing and over-detailed explanatory text while preserving full transparency regarding variable categorization, regression modeling strategy, and statistical thresholds. | Methods—Statistical analyses: Page 12, Lines 241–261 and Page 13, Lines 262–265. |
| **Reviewer 2 Comment 9: (– Results Tables):**  Tables should be reviewed by a biostatistician and possibly merged. | **Response:**  Thank you for this constructive suggestion. In consultation with a biostatistician, we determined that merging Tables would reduce clarity, as these tables present results from distinct populations (dentists vs. patients) and different outcome measures (continuous attitude scores vs. dichotomized attitude domains vs. willingness to quit). Combining them would have made interpretation more difficult for readers. Thus, to streamline the presentation while preserving the integrity of the data, we have: • Moved Table 2 (bivariate associations for dentists) to a supplementary file (S1_Table), as this table primarily provides descriptive bivariate comparisons that support but are secondary to the main regression findings presented in the text and could have caused confusion in interpreting the results. • Retained the other Tables the main manuscript, as they present infromation on the demographic makeup of the sample or results from regression analyses that address the study's primary analytical objectives. • Updated the Results section to reflect this change, with a concise summary of the moved table's key findings and a clear reference to the supplementary file. • Moved the text describing Table 2 to the section on predictors of dentist attitudes in a way that it precedes the presentation of predictors. This was done to more clearly depict the statistical pathway taken and avoid confusing the reader by spreading these findings all over the manucsript. We believe these revisions improve the flow and focus of the main results while ensuring all data remain fully accessible to readers. | Results—Individual-level determinants of dentists’ SCCD attitudes: Page 19, Lines 336–341 and Page 20, Lines 342–346. Supporting information list, including S1 Table: Page 33, Lines 632–638. |
| **Reviewer 2 Comment 10: (– Discussion):**  Discussion is overly lengthy; insufficient consideration of divergent evidence. | Response (A) Length: We thank the reviewer for highlighting this issue. The Discussion has been condensed as much as possible through both structural and content-level revisions. In the previous version, length was driven by repetition and parallel presentation of similar findings across stakeholder groups.  In the revised version, organizing the Discussion around shared determinants across stakeholders enabled consolidation of overlapping content and removal of redundant explanations, resulting in a more concise and focused narrative.  Changes made: • Merged overlapping dentist and patient discussions into unified determinant-based paragraphs:  o smoking status  o employment • Removed duplicated conceptual blocks:  o repeated dentist–patient contrast eliminated • Condensed key paragraphs:  o non-smoking  o employment  o contextual determinants • Reduced citation density and eliminated redundancy across Discussion  (B) Divergent Evidence Response: We appreciat the reviewer’s point and have substantially strengthened engagement with divergent evidence. In the previous version, the Discussion largely presented confirmatory findings with limited critical comparison. In the revised version, divergent evidence is systematically incorporated within each determinant-focused paragraph, ensuring balanced and critical synthesis across all key findings. Each paragraph now includes supporting evidence, contrasting findings (where available), and explicit explanations for discrepancies based on methodological and contextual differences.  Changes made: • For non-smoking, employment, and contextual determinants:  o Added contradictory evidence  o Explained potential reasons for the contradictions | Discussion—condensed determinant-based structure and divergent evidence: Page 20, Lines 348–375; Page 21, Lines 376–386; Page 22, Lines 387–409; Page 23, Lines 410–432; Page 24, Lines 433–448. |
| **Reviewer 2 Comment 11: (– Discussion):**  Insufficient consideration of divergent evidence. | **Response:** We appreciat the reviewer’s point and have substantially strengthened engagement with divergent evidence. In the previous version, the Discussion largely presented confirmatory findings with limited critical comparison. In the revised version, divergent evidence is systematically incorporated within each determinant-focused paragraph, ensuring balanced and critical synthesis across all key findings. Each paragraph now includes supporting evidence, contrasting findings (where available), and explicit explanations for discrepancies based on methodological and contextual differences.  **Changes made:** • For non-smoking, employment, and contextual determinants:  o Added contradictory evidence  o Explained potential reasons for the contradictions | Discussion—divergent/cautious interpretation: Page 21, Lines 384–386; Page 22, Lines 387–401 and 407–413; Page 23, Lines 418–432; Page 24, Lines 433–448. |
| **Reviewer 2 Comment 12: (– Discussion):**  Add a dedicated “Limitations” subsection. | **Response:** Thank you for this helpful suggestion. We have added a dedicated “Strengths and Limitations” subsection to the Discussion section. This subsection explicitly outlines the methodological  limitations of the study. We have also clarified these factors should be considered when interpreting the findings.  **Change Made:** A dedicated “Strengths and Limitations” subsection has been added to the Discussion section. | Discussion—Strengths and Limitations subsection: Page 24, Lines 449–454 and Page 25, Lines 455–466. Related implications/future directions following the subsection: Page 25, Lines 467–478 and Page 26, Lines 479–484. |
