# Supplementary material for: Smoking cessation counseling in primary care settings: Cross-sectional views from patients and dentists as key stakeholders
Source: PLoS One. 2026 Aug 14;21(8):e0354366. doi: 10.1371/journal.pone.0354366 (PMC13475981; doi:10.1371/journal.pone.0354366)
Supplement: Peer review history

# Peer review: Decision Letter 0

**DOI:** 10.1371/journal.pone.0354366.r001

**Authors:** Fatemeh Zarei

13 Feb 2026

Dear Dr. Khami,

Thank you for submitting your manuscript to PLOS ONE. After careful consideration, we feel that it has merit but does not fully meet PLOS ONE’s publication criteria as it currently stands. Therefore, we invite you to submit a revised version of the manuscript that addresses the points raised during the review process.

Please submit your revised manuscript by Mar 30 2026 11:59PM l. If you will need more time than this to complete your revisions, please reply to this message or contact the journal office at plosone@plos.org. When you're ready to submit your revision, log on to https://www.editorialmanager.com/pone/ and select the 'Submissions Needing Revision' folder to locate your manuscript file.

- A letter that responds to each point raised by the academic editor and reviewer(s). You should upload this letter as a separate file labeled 'Response to Reviewers'.
- A marked-up copy of your manuscript that highlights changes made to the original version. You should upload this as a separate file labeled 'Revised Manuscript with Track Changes'.
- An unmarked version of your revised paper without tracked changes. You should upload this as a separate file labeled 'Manuscript'.

If you would like to make changes to your financial disclosure, please include your updated statement in your cover letter. Guidelines for resubmitting your figure files are available below the reviewer comments at the end of this letter.

If applicable, we recommend that you deposit your laboratory protocols in protocols.io to enhance the reproducibility of your results. Protocols.io assigns your protocol its own identifier (DOI) so that it can be cited independently in the future. For instructions see: https://journals.plos.org/plosone/s/submission-guidelines#loc-laboratory-protocols. Additionally, PLOS ONE offers an option for publishing peer-reviewed Lab Protocol articles, which describe protocols hosted on protocols.io. Read more information on sharing protocols at https://plos.org/protocols?utm_medium=editorial-email&utm_source=authorletters&utm_campaign=protocols.

We look forward to receiving your revised manuscript.

Kind regards,

Fatemeh Zarei, PhD

Academic Editor

PLOS One

Journal Requirements:

When submitting your revision, we need you to address these additional requirements.

1.Please ensure that your manuscript meets PLOS ONE's style requirements, including those for file naming. The PLOS ONE style templates can be found at

https://journals.plos.org/plosone/s/file?id=wjVg/PLOSOne_formatting_sample_main_body.pdf and

https://journals.plos.org/plosone/s/file?id=ba62/PLOSOne_formatting_sample_title_authors_affiliations.pdf

2. We note that the grant information you provided in the ‘Funding Information’ and ‘Financial Disclosure’ sections do not match.

When you resubmit, please ensure that you provide the correct grant numbers for the awards you received for your study in the ‘Funding Information’ section.

3. Thank you for stating the following financial disclosure:

“M.K received partial financial assistance for  this study from Tehran Univresity of Medical Sciences”

Please state what role the funders took in the study.  If the funders had no role, please state: "The funders had no role in study design, data collection and analysis, decision to publish, or preparation of the manuscript."

If this statement is not correct you must amend it as needed.

Please include this amended Role of Funder statement in your cover letter; we will change the online submission form on your behalf.

4. We note that there is identifying data in the Supporting Information file “S4_file.xlsx, S1_file.docx and S3_file.xlsx”. Due to the inclusion of these potentially identifying data, we have removed this file from your file inventory. Prior to sharing human research participant data, authors should consult with an ethics committee to ensure data are shared in accordance with participant consent and all applicable local laws.

Data sharing should never compromise participant privacy. It is therefore not appropriate to publicly share personally identifiable data on human research participants. The following are examples of data that should not be shared:

-Name, initials, physical address

-Ages more specific than whole numbers

-Internet protocol (IP) address

-Specific dates (birth dates, death dates, examination dates, etc.)

-Contact information such as phone number or email address

-Location data

-ID numbers that seem specific (long numbers, include initials, titled “Hospital ID”) rather than random (small numbers in numerical order)

Data that are not directly identifying may also be inappropriate to share, as in combination they can become identifying. For example, data collected from a small group of participants, vulnerable populations, or private groups should not be shared if they involve indirect identifiers (such as sex, ethnicity, location, etc.) that may risk the identification of study participants.

Additional guidance on preparing raw data for publication can be found in our Data Policy (https://journals.plos.org/plosone/s/data-availability#loc-human-research-participant-data-and-other-sensitive-data) and in the following article: http://www.bmj.com/content/340/bmj.c181.long.

Please remove or anonymize all personal information (<specific identifying information in file to be removed>), ensure that the data shared are in accordance with participant consent, and re-upload a fully anonymized data set. Please note that spreadsheet columns with personal information must be removed and not hidden as all hidden columns will appear in the published file.

5. If the reviewer comments include a recommendation to cite specific previously published works, please review and evaluate these publications to determine whether they are relevant and should be cited. There is no requirement to cite these works unless the editor has indicated otherwise.

Additional Editor Comments:

After a thorough review of your manuscript, "Smoking cessation counseling in primary care settings," both reviewers have identified several critical areas that require your attention to elevate the work to a publishable standard. The consensus indicates that while the topic is significant, the **methodological transparency**  and **literature currency**  are currently insufficient. Specifically, you must update your introduction with references from the last  recent years(NOT ONLY 4 YEARS) to reflect the modern epidemiological landscape and clarify the study design (e.g., descriptive vs. analytical). Furthermore, the reviewers highlighted a lack of detail regarding the **validity, reliability, and psychometric properties**  of your research instruments, which is essential for establishing the credibility of your findings. Addressing these points along side refining the statistical reporting and streamlining the discussion is necessary to ensure the manuscript provides a cohesive, rigorous, and up-to-date contribution to the field of primary care

[Note: HTML markup is below. Please do not edit.]

Reviewer's Responses to Questions

**Comments to the Author**

1. Is the manuscript technically sound, and do the data support the conclusions?

Reviewer #1: Partly

Reviewer #2: Yes

**********

2. Has the statistical analysis been performed appropriately and rigorously?

Reviewer #1: Yes

Reviewer #2: Yes

**********

3. Have the authors made all data underlying the findings in their manuscript fully available?

The PLOS Data policy

Reviewer #1: Yes

Reviewer #2: Yes

**********

4. Is the manuscript presented in an intelligible fashion and written in standard English?

Reviewer #1: Yes

Reviewer #2: Yes

**********

Reviewer #1: Abstract

The method should specify the type of cross-sectional study: descriptive or analytical?

Keywords should be revised and adjusted to match the title. Many terms are not even in the abstract.

Introduction

Sources 1 to 6 are old and unreliable. Very new sources are needed for epidemiology.

In the first paragraph, the references are not in order.

The introduction lacks detailed and up-to-date background and is written more like an epidemiology.

Method

Confidentiality is mentioned once in the ethical principles and once again in the procedure, avoiding repetition of issues.

The information in the instrument is incomplete. What is the scoring method?

The validity and reliability method are not clear.

How is the validity and reliability of the original questionnaire?

The source is not reliable.

The terms dependent and independent variables belong to the experimental method. It is better to provide an alternative term.

Once in the tool section, the tool is given in general terms and once in the dependent variable. The reader is confused. These sections should be written more coherently.

Statistical analyses should be written more concisely. Descriptive information is not included in the introduction to this section and is repeated in Table 1.

Based on the findings, the research method is a descriptive cross-sectional study. This issue has not been mentioned anywhere.

Several comparisons with the research literature are written in detail in the discussion, which makes the reader unaware of the findings of the literature. These findings should come in introduction, and comparisons are discussed here. So, in this section, the comparisons will be summarized and the distinguishing feature of this study should be explained.

Most of the sources cited as research background are old and should be from the last 4 years.

Reviewer #2: Dear Editor-in-Chief of PLoS ONE,

Please find below my feedback on the manuscript titled, "Smoking cessation counseling in primary care settings: cross-sectional views from patients and dentists as key stakeholders."

Abstract

The title and abstract are well-structured. However, it is recommended that the terminology be verified against MeSH (Medical Subject Headings) for optimal indexing.

Introduction

Several references cited to establish prevalence and incidence (e.g., references 1, 2, and 5) are outdated and should be replaced with the most current literature. Furthermore, sentences supported by multiple citations (particularly on pages 4 and 5) should be reviewed to ensure all referenced sources demonstrate consensus on the stated claims.

While the cited literature is relevant, the narrative lacks cohesiveness, which obscures the articulation of the research gap and the study's rationale. The introduction would benefit from a clearer delineation of the research significance and necessity.

Methods

The rationale for restricting participant selection to individuals over 18 years of age is insufficiently explained.

The description of the study sample mentions the numbers 163, 188, and 367 (page 7), which requires clarification.

The development process, validation history, permissions for use, and psychometric properties of the employed instrument are not transparently detailed (page 8). Providing this information is crucial; focusing predominantly on the scoring guide is inadequate.

The statistical analysis section (pages 10 and 11) is excessively detailed and could be condensed without losing essential information.

Results

The findings are comprehensively presented in tables aligned with the study objectives. It is recommended that these tables be reviewed and validated by a biostatistician. Some tables might be candidates for merging to improve conciseness and clarity.

Discussion

The discussion is overly lengthy and would benefit from condensation. It primarily adopts a convergent narrative, with insufficient consideration of divergent perspectives or contradictory evidence. Additionally, many statements are supported by multiple references; it is essential to confirm that all cited sources agree with the presented claims.

A dedicated "Limitations" subsection should be added to the manuscript.

**********

what does this mean?). If published, this will include your full peer review and any attached files.

If you choose “no”, your identity will remain anonymous but your review may still be made public.

**Do you want your identity to be public for this peer review?**  For information about this choice, including consent withdrawal, please see our Privacy Policy

Reviewer #1: **Yes:** Fatteme Raiisi

Reviewer #2: **Yes:** Fazlollah Ahmadi

**********

[NOTE: If reviewer comments were submitted as an attachment file, they will be attached to this email and accessible via the submission site. Please log into your account, locate the manuscript record, and check for the action link "View Attachments". If this link does not appear, there are no attachment files.]

To ensure your figures meet our technical requirements, please review our figure guidelines: https://journals.plos.org/plosone/s/figures

You may also use PLOS’s free figure tool, NAAS, to help you prepare publication quality figures: https://journals.plos.org/plosone/s/figures#loc-tools-for-figure-preparation.

NAAS will assess whether your figures meet our technical requirements by comparing each figure against our figure specifications.

Attachment Submitted filename: Abstract.docx

# Author comment: Author response to Decision Letter 1

**DOI:** 10.1371/journal.pone.0354366.r002

25 Apr 2026

Uploaded in the attach files section as it was too lengthy to copy and paste here.

Attachment Submitted filename: point-by-point response-plos one-round 1.pdf

# Peer review: Decision Letter 1

**DOI:** 10.1371/journal.pone.0354366.r003

**Authors:** Fatemeh Zarei

12 May 2026

PLOS One

Dear Dr. Khami,

Thank you for submitting your manuscript to PLOS ONE. After careful consideration, we feel that it has merit but does not fully meet PLOS ONE’s publication criteria as it currently stands. Therefore, we invite you to submit a revised version of the manuscript that addresses the points raised during the review process.

==============================

**Comment to the author:**

Your response letter is currently difficult to follow and unsuitable for proper judgment. Please revise the format so that **each reviewer comment is addressed separately**, and for every response, clearly indicate the exact location (page and line number) of the corresponding changes in the revised manuscript.

Use the following structure:

**Reviewer 1**

Comment 1: …

Response 1: … (Page X, Line Y)

**Reviewer 2**

Comment 1: …

Response 1: … (Page X, Line Y)

Do not combine multiple comments or responses into one paragraph. Mark every change explicitly. Resubmit the response letter accordingly.

Provide a new answer letter for all previous and new comments at the end of your manuscript. It's recommended to illustrate it in a table

==============================

Please submit your revised manuscript by Jun 26 2026 11:59PM. If you will need more time than this to complete your revisions, please reply to this message or contact the journal office at plosone@plos.org. When you're ready to submit your revision, log on to https://www.editorialmanager.com/pone/ and select the 'Submissions Needing Revision' folder to locate your manuscript file.

Please include the following items when submitting your revised manuscript:

- A letter that responds to each point raised by the academic editor and reviewer(s). You should upload this letter as a separate file labeled 'Response to Reviewers'.
- A marked-up copy of your manuscript that highlights changes made to the original version. You should upload this as a separate file labeled 'Revised Manuscript with Track Changes'.
- An unmarked version of your revised paper without tracked changes. You should upload this as a separate file labeled 'Manuscript'.

If you would like to make changes to your financial disclosure, please include your updated statement in your cover letter. Guidelines for resubmitting your figure files are available below the reviewer comments at the end of this letter.

If applicable, we recommend that you deposit your laboratory protocols in protocols.io to enhance the reproducibility of your results. Protocols.io assigns your protocol its own identifier (DOI) so that it can be cited independently in the future. For instructions see: https://journals.plos.org/plosone/s/submission-guidelines#loc-laboratory-protocols. Additionally, PLOS ONE offers an option for publishing peer-reviewed Lab Protocol articles, which describe protocols hosted on protocols.io. Read more information on sharing protocols at https://plos.org/protocols?utm_medium=editorial-email&utm_source=authorletters&utm_campaign=protocols.

As the corresponding author, your ORCID iD is verified in the submission system and will appear in the published article. PLOS supports the use of ORCID, and we encourage all coauthors to register for an ORCID iD and use it as well. Please encourage your coauthors to verify their ORCID iD within the submission system before final acceptance, as unverified ORCID iDs will not appear in the published article. *Only*  the individual author can complete the verification step; PLOS staff *cannot*  verify ORCID iDs on behalf of authors.

We look forward to receiving your revised manuscript.

Kind regards,

Fatemeh Zarei, PhD

Academic Editor

PLOS One

Journal Requirements:

1. If the reviewer comments include a recommendation to cite specific previously published works, please review and evaluate these publications to determine whether they are relevant and should be cited. There is no requirement to cite these works unless the editor has indicated otherwise.

2. Please review your reference list to ensure that it is complete and correct. If you have cited papers that have been retracted, please include the rationale for doing so in the manuscript text, or remove these references and replace them with relevant current references. Any changes to the reference list should be mentioned in the rebuttal letter that accompanies your revised manuscript. If you need to cite a retracted article, indicate the article’s retracted status in the References list and also include a citation and full reference for the retraction notice.

[Note: HTML markup is below. Please do not edit.]

Reviewers' comments:

Reviewer's Responses to Questions

**Comments to the Author**

Reviewer #1: All comments have been addressed

Reviewer #2: All comments have been addressed

**********

2. Is the manuscript technically sound, and do the data support the conclusions?

Reviewer #1: Yes

Reviewer #2: Yes

**********

3. Has the statistical analysis been performed appropriately and rigorously?

Reviewer #1: Yes

Reviewer #2: Yes

**********

4. Have the authors made all data underlying the findings in their manuscript fully available?

The PLOS Data policy

Reviewer #1: Yes

Reviewer #2: Yes

**********

5. Is the manuscript presented in an intelligible fashion and written in standard English?

Reviewer #1: (No Response)

Reviewer #2: Yes

**********

Reviewer #1: What is the reason for referencing in the setting section?

The validity and reliability of the questionnaires have not been stated.

Instead of World Health Organization references, provide more accurate and up-to-date references.

Many of the sources are outdated.

Reviewer #2: No further comments left, I prefer the final decision be based on editor comments. Thanks for considering suggestion

**********

what does this mean?). If published, this will include your full peer review and any attached files.

If you choose “no”, your identity will remain anonymous but your review may still be made public.

**Do you want your identity to be public for this peer review?**  For information about this choice, including consent withdrawal, please see our Privacy Policy

Reviewer #1: **Yes:**

Reviewer #2: **Yes:** Fazlollah Ahmadi

**********

[NOTE: If reviewer comments were submitted as an attachment file, they will be attached to this email and accessible via the submission site. Please log into your account, locate the manuscript record, and check for the action link "View Attachments". If this link does not appear, there are no attachment files.]

To ensure your figures meet our technical requirements, please review our figure guidelines: https://journals.plos.org/plosone/s/figures

You may also use PLOS’s free figure tool, NAAS, to help you prepare publication quality figures: https://journals.plos.org/plosone/s/figures#loc-tools-for-figure-preparation.

NAAS will assess whether your figures meet our technical requirements by comparing each figure against our figure specifications.

# Author comment: Author response to Decision Letter 2

**DOI:** 10.1371/journal.pone.0354366.r004

1 Jul 2026

The responses to all comments have been provided in the response to reviewers' file that has been uploaded.

Attachment Submitted filename: Response_to_Academic_Editor_and_Reviewers_round2_.docx

# Editor report: Decision Letter 2

**DOI:** 10.1371/journal.pone.0354366.r005

**Authors:** Fatemeh Zarei

7 Jul 2026

Smoking cessation counseling in primary care settings: cross-sectional views from patients and dentists as key stakeholders

PONE-D-25-68934R2

Dear Dr. Mohammad Reza Khami

We’re pleased to inform you that your manuscript has been judged scientifically suitable for publication and will be formally accepted for publication once it meets all outstanding technical requirements.

Within one week, you’ll receive an e-mail detailing the required amendments. When these have been addressed, you’ll receive a formal acceptance letter and your manuscript will be scheduled for publication.

An invoice will be generated when your article is formally accepted. Please note, if your institution has a publishing partnership with PLOS and your article meets the relevant criteria, all or part of your publication costs will be covered. Please make sure your user information is up-to-date by logging into Editorial Manager at Editorial Manager® and clicking the ‘Update My Information' link at the top of the page. For questions related to billing, please contact billing support.

If your institution or institutions have a press office, please notify them about your upcoming paper to help maximize its impact. If they’ll be preparing press materials, please inform our press team as soon as possible -- no later than 48 hours after receiving the formal acceptance. Your manuscript will remain under strict press embargo until 2 pm Eastern Time on the date of publication. For more information, please contact onepress@plos.org.

Kind regards,

Fatemeh Zarei, PhD

Academic Editor

PLOS One

Additional Editor Comments (optional):

Reviewers' comments:

# Editor report: Acceptance letter

**DOI:** 10.1371/journal.pone.0354366.r006

**Authors:** Fatemeh Zarei

PONE-D-25-68934R2

PLOS One

Dear Dr. Khami,

I'm pleased to inform you that your manuscript has been deemed suitable for publication in PLOS One. Congratulations! Your manuscript is now being handed over to our production team.

At this stage, our production department will prepare your paper for publication. This includes ensuring the following:

* All references, tables, and figures are properly cited

* All relevant supporting information is included in the manuscript submission,

* There are no issues that prevent the paper from being properly typeset

You will receive further instructions from the production team, including instructions on how to review your proof when it is ready. Please keep in mind that we are working through a large volume of accepted articles, so please give us a few days to review your paper and let you know the next and final steps.

Lastly, if your institution or institutions have a press office, please let them know about your upcoming paper now to help maximize its impact. If they'll be preparing press materials, please inform our press team within the next 48 hours. Your manuscript will remain under strict press embargo until 2 pm Eastern Time on the date of publication. For more information, please contact onepress@plos.org.

You will receive an invoice from PLOS for your publication fee after your manuscript has reached the completed accept phase. If you receive an email requesting payment before acceptance or for any other service, this may be a phishing scheme. Learn how to identify phishing emails and protect your accounts at https://explore.plos.org/phishing.

If we can help with anything else, please email us at customercare@plos.org.

Thank you for submitting your work to PLOS ONE and supporting open access.

Kind regards,

PLOS ONE Editorial Office Staff

on behalf of

Dr. Fatemeh Zarei

Academic Editor

PLOS One
